# Consumers’ Attitudes and Preferences Towards Ingredients List, Nutrition Information and Health Warning Labelling on Alcohol Products: A Scoping Review

**DOI:** 10.1007/s13668-026-00784-y

**Published:** 2026-07-22

**Authors:** Milana Crevar, Adrian Esterman, Armando Maria Corsi, Vicki Waye, Colin Ireland

**Affiliations:** 1 School of Public Health, Adelaide University, City Campus East, GPO Box 2471, Adelaide, SA 5001 Australia; 2 Adelaide Business School, The University of Adelaide, 10 Pulteney Street, Adelaide, SA 5005 Australia; 3 College of Business and Law, Adelaide University, City Campus East, GPO Box 2471, Adelaide, SA 5001 Australia; 4 College of Health, School of Nursing and Midwifery, Adelaide University, City Campus East, GPO Box 2471, Adelaide, SA 5001 Australia

**Keywords:** Alcohol, Consumer, Health warning, Ingredient list, Label, Nutrition information

## Abstract

**Purpose of Review:**

One way to reduce the health hazards of excess alcohol consumption and the subsequent health concerns is to include information on the label. While alcoholic beverages are characterised as food, they are exempt from some of the labelling information and requirements that apply to other food items, including not requiring total ingredient lists or nutrition information. A scoping review following a systematic process was undertaken using the Preferred Reporting Items for Systematic Reviews and Meta-Analyses extension for Scoping Reviews. This scoping review focuses on consumer preferences and attitudes relating to ingredient lists, nutrition information, and health warnings on alcohol labels. 56 papers were included in the review.

**Recent Findings:**

We found that attitudes and preferences towards alcohol labels are mixed, and while consumers express interest in nutrition, ingredients and health warning information, their actual use of this information for decision-making remains limited. Those who are most likely to use information on labels, and think that the labels would influence their alcohol consumption are females, lighter drinkers and those who are more health conscious.

**Summary:**

Consumer attitudes and preferences towards alcohol labels are mixed. Consumer preferences influence and play an important role in determining acceptance of labels and what consumers want. This review has identified gaps in the literature around consumer preferences for nutrition and ingredients listings, while there is an abundance of evidence relating to health warnings on alcohol labels with mixed results.

**Supplementary Information:**

The online version contains supplementary material available at 10.1007/s13668-026-00784-y.

## Introduction

Alcohol has been widely used for centuries [1], but according to the World Health Organization (WHO), harmful alcohol consumption is the third highest contributor to disease and disability, accounting for 4.7% of the global burden of disease [2]. Alcohol consumption is associated with elevated risks of cardiovascular disease, cancer, liver disease, and obesity [2–5]. As a psychoactive drug, excess alcohol consumption contributes to social problems and violent crime [6, 7].

A range of policy strategies has been recommended to reduce alcohol-related harm, including taxation, restrictions on availability and marketing, and alcohol labelling measures endorsed in WHO guidance [8, 9]. One such strategy is to provide health-related information on alcohol packaging, similar to the warning labels used on tobacco products around the world for many years [10]. Several countries mandate alcohol warning labels, including general warnings in South Africa [11] and drink-driving warnings in the United States [12], Turkey [13], Thailand [14], and Mexico [15]. Pregnancy warnings are mandatory in Australia and New Zealand [16], the United States [12], Ireland [17], and France [18]. South Korea [19] and Ireland [20] also require cancer-related health warnings on alcohol labels.

However, only 32.5% of WHO Member States mandate alcohol health warning labels [21]. Several countries rely on voluntary consumer health messaging or supplement mandatory warnings with additional voluntary warnings. Examples of countries which mainly rely on voluntary messaging include Canada [21] and the United Kingdom [22]. In Australia, mandatory pregnancy warnings are supplemented by voluntary messaging activity undertaken by DrinkWise [23], established by government and industry in 2005, and entirely industry-funded since 2009 [23, 24]. Examples of DrinkWise messaging include: ‘Is Drinking Harming Yourself or Others’ and ‘Kids and Alcohol Don’t Mix’ [25]. While these organisations promote responsible drinking message, they have been criticised for shifting responsibility for the alcohol related harm onto consumers [26]. Critics also argue that this messaging is largely ineffective [24] and that industry bodies promoting moderate drinking actually serve as front groups for social marketing and public relations activities on behalf of risk industries [27].

Despite alcohol being classified and regulated as a food product in many jurisdictions including Australia and New Zealand [16, 28, 29], there is no uniform international alcohol labelling standard [30]. Most countries require labelling to state basic information such as lot identification, name of supplier, and alcohol by volume, but many such as the United States, the United Kingdom and Australia do not require nutritional or ingredients labelling [31]. This creates a paradox: alcohol faces strict regulation yet avoids standard food information requirements [32].

As better labelling could enable informed choices affecting health outcomes, advocates believe consumers deserve the same information for alcoholic beverages as other food products [33, 34]. As requirements for health warnings, ingredient and nutrition information differ across countries these variations have resulted in inconsistent consumer access to transparent information on packaged alcoholic beverages. International pressure is therefore growing to strengthen alcohol labelling requirements [8, 35]. Australia and New Zealand have implemented mandatory energy labelling and its tabular label format [16, 31], while the European Union requires energy disclosure on wine labels with optional online nutrition and ingredients information [36]. Ireland mandates calorie content and explicit health warnings [17, 20].

Consumer attitudes toward food labelling can significantly affect purchasing decisions [37–41]. Understanding consumer perspectives on alcohol labelling helps guide effective policy design and implementation. This scoping review aims to establish understanding of consumer attitudes and preferences regarding nutrition, ingredient and health warning labels on alcohol products, assessing consumer awareness, attention and understanding of this information.

## Methodology

A scoping review following Arksey and O’Malley’s [42] systematic process was undertaken to understand consumer preferences and attitudes regarding nutrition, ingredient and health warning labelling of alcohol products. We used the Preferred Reporting Items for Systematic Reviews and Meta-Analyses extension for Scoping Reviews (PRISMA-ScR), checklist guided reporting (Appendix 1) [43]. A review protocol was not registered. To guide the review the population, concept and context framework (PCC) was used [44]. Population: general public; Concept: consumer preferences and attitudes towards nutrition, ingredient, and health warning labels on alcohol, as well as awareness, attention and understanding; Context: all countries where the reports were written in English.

### Research Questions

This review addressed three questions. First, how much awareness and attention do consumers have towards nutrition, ingredients and health warning labels on alcoholic beverages, and how much do consumers understand these labels. Second, what are consumers’ attitudes regarding content and preferences for alcohol label design—nutrition, ingredients and health warnings. Third, how might consumers’ awareness and attitudes towards nutrition, ingredients and health warning labels on alcoholic beverages affect their intention to purchase.

### Search Strategy

Search terms were developed with academic librarian assistance according to Medical Subject Headings (MeSH) and specific keywords. Electronic databases searched included ABI/Inform collection, Informit Business Collection, EBSCohost Business Source Complete, Emerald Insight, ProQuest central, ProQuest dissertation & theses global, Scopus, Web of Science, Ovid Medline, Ovid Embase and Ovid Emcare, plus Google Scholar. We provide full details of search terms used in Appendix 2.

Inclusion criteria included studies reporting consumer attitudes and preferences on nutrition information, ingredients listings and health warnings on alcohol labelling, written in English, published 2010–2020. Reported behavioural responses were captured where studies reported them. Review papers and opinion pieces were excluded to minimise duplication.

### Study Selection and Reporting Guidelines Assessment

Endnote X8 managed search results, with data uploaded to Covidence^®^ for screening. Two reviewers independently screened titles, abstracts and full texts, with conflicts resolved by a third reviewer. The authors assessed reporting quality using the Strengthening the Reporting of Observational Studies in Epidemiology (STROBE) Statement outline [45]. The STROBE Statement comprising 22 checklist items was applied to the “title, abstract, introduction, methods, results and discussion sections of studies” [45]. For randomised controlled trials, the Consolidated Standards of Reporting Trials (CONSORT) was applied, and the CONSORT 2010 checklist was used comprising the 25 checklist items that relate to the “title and abstract, introduction, methods, results, discussion and other information” [46]. The STROBE Statement and CONSORT 2010 checklists helped to assess reporting completeness. Each checklist item was allocated one point.

The reporting quality assessment of the included studies was undertaken and presented using a modified traffic light system utilised in Cochrane reviews [47]. The colours show the level of reporting quality, ranging from low (red) to high (green). For the observational studies (STROBE checklist) the following was used 1–7 points, red light, 8–14 points yellow light, and 15–22 points green light. Randomised control studies (CONSORT checklist) scores of 1–8 red light, 9–16 yellow light and 17–25 green light were used.

## Results

### Study Selection

**Fig. 1:**
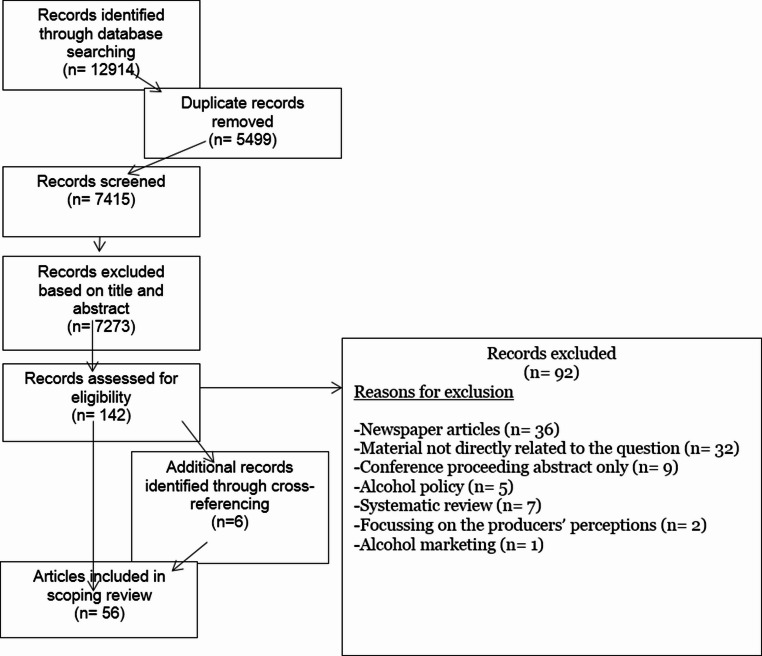
Prisma diagram of scoping review

Following PRISMA-ScR (Fig. 1) flow chart [43], 12,914 studies were imported for initial screening. After removing duplicates and screening, 50 articles remained, with six additional articles identified from reference lists. A total of 56 studies were included: 18 on nutrition information labelling (Appendix 3), six on ingredients list labelling (Appendix 4), and 45 on health warning labels (Appendix 5).

### Study Assessment

**Fig. 2:**
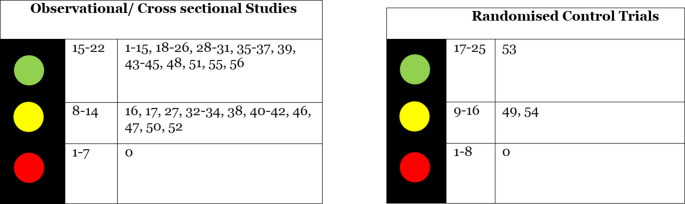
The modified traffic light system

The 53 observational and mixed methods studies received quality assessment scores between 10 and 21, with three randomised control trials scoring 15, 16 and 18 (Fig. 2). Overall, 39 studies received green light ratings (15–22) showing medium to high quality, while 14 received yellow light ratings (8–14). Common omissions included failure to report bias sources, statistical methods, sensitivity analyses, and generalisability discussions.

### Study Characteristics

Most participants were aged 18 years or older, with 31 studies requiring alcohol consumption and 17 targeting heavy/high-risk drinkers. Recruitment sources included market research agencies (*n* = 17), universities (*n* = 16), and online recruitment (*n* = 6). Female participants were overrepresented, especially in health warning studies.

Thirty-three were observational/cross-sectional studies, 20 had experimental and mixed methods designs, and three were randomised control trials. Most research (*n* = 50) was published in 2015 or later, predominantly in Western countries (*n* = 55), with the United Kingdom (*n* = 13) and Australia (*n* = 12) leading.

### Consumer Awareness, Attention and Understanding

#### Awareness

Consumer awareness of nutrition labels was connected to nutritional knowledge [48, 49]. Consumers were asked to estimate the kcal content of a glass of wine and to identify which alcoholic beverage contains the highest calorie content [48, 50]; only 22% of consumers were aware of wine glass calorie content [48], and 34% knew alcopops had highest calorie content [50]. Consumers were also unaware that alcohol itself is the main energy source, believing it was sugar [51].

For health warnings, most studies found little consumer awareness [52–56], though consumers were aware of warnings about alcohol abuse, drink driving and pregnancy risks covered in prior health campaigns [55, 57, 58].

#### Attention

Back labels played minor roles in decision-making, with price, bottle design, occasion or recommendations more influential than nutrition panels, calories, ingredients and health warning messages [59, 60]. Only one-third of consumers examining back labels detected new ingredients and nutrition information, with most declaring their buying behaviour wouldn’t change long-term [59].

If ingredients and nutrition information was made available, three-quarters of consumers reported that they would use brand labels or websites to find and read this information [61]. Studies concluded that gaining consumer attention efficiently shouldn’t rely on one medium but should incorporate multiple sources including public campaigns, signs, digital platforms, posters, QR codes, advertisements, and other media [52, 53, 57, 58, 60–69].

Consumer attention to nutrition information was related to their ability to understand this information [48]. Those paying more attention had easier understanding of content like calorie identification [48, 50, 59].

For ingredients, negative media coverage increased attention to ingredients labelling [70]. Consumers reported they paid more attention when negative media information about wine ingredients was published, influencing their preferences [70].

Consumers reported paying little attention to health warning labels due to low noticeability and credibility issues [52, 54–57, 60, 62, 71, 72]. Eye-tracking found consumers spent only 7% of viewing time on health warning labels [72], with recall rates at best 16% [53].

#### Understanding

Consumers expressed limited understanding of nutrition panels, calories and energy information for alcoholic beverages [48–51, 58, 59, 73]. Confusion existed about calories and energy content [51, 59, 73]. Consumers underestimated presented calories by 43%, with 17% believing wine contained no calories [50], while other studies found overestimation [58, 59].

Limited understanding existed for ingredients information [59]. Two out of three consumers didn’t understand provided ingredients information, viewing wine as very natural with no ingredients except grapes [59, 70].

For health warnings, unprompted recall of voluntary pregnancy warning labels was 3% in New Zealand [74]. When prompted visually, 44% recalled at least one pregnancy warning label with clear understanding of meaning and pregnancy drinking risks [74]. Health warnings around fertility, mental health and cancer provided new information to consumers [58, 69, 75].

### Consumer Attitudes

#### Positive Attitudes

The studies reported that consumers wanted nutrition, energy, calorie, and ingredient information for alcoholic beverages on labels [48, 50, 51, 58, 63, 73, 76–79]. A study of 9,008 consumers found 86% believe they should have access to the same nutrition and ingredients information for alcoholic beverages as other food and drink products [61].

Consumers in several countries stated nutrition information on wine labels would affect their wine choice [70]. Martinez et al. [77] found 85% of consumers wanted nutrition information. Support existed for mandatory ingredients labelling, particularly for allergic reactions and production transparency [59, 78].

Similarly the studies concluded that evidence-based alcohol health warnings like tobacco could affect consumer perceptions [80]. Warning labels may raise awareness about links between serious diseases and regular alcohol consumption [65, 67, 75]. Some studies reported warning labels increased consumer knowledge levels and reduced drinking [65, 81–83].

#### Negative Attitudes and Reported Behavioural Responses

Despite support for mandatory nutrition, energy, and ingredients information, consumers declared that their buying behaviour wouldn’t change long-term [51, 59, 79]. Many studies supported consumers’ lack of interest based on minimal effect on drinking behaviour [49, 51, 59, 64, 73, 77, 79].

For health warnings, many studies concluded that while warnings could be effective, they were unlikely to change respondents’ drinking behaviour [54, 55, 57, 62, 64, 66, 72, 84, 85]. However, negative attitudes were also reported, with over a quarter of consumers stating that they would try to avoid health warnings, finding them annoying and controlling [55, 57, 58].

#### Consumption Frequency and Demographics

Across studies, consumers classified by the original authors as moderate consumption consumers viewed health warnings positively, while higher consumption consumers showed least interest [48, 52, 58, 67, 76, 86]. Higher alcohol consumption frequency consumers were less likely to comply with recommendations and showed lower awareness of alcohol related harms and weaker attitudes towards label warnings [52, 55, 67, 87]. Light to moderate drinkers were more interested in nutrition panel, energy and ingredient information than heavy drinkers [51, 61]. However, definitions of consumption categories varied across studies.

Older consumers were more interested in ingredients information but demonstrated limited nutrition knowledge [88]. They were more likely to notice wine labels, read warnings, and reported willingness to adjust behaviour accordingly [63, 67]. Younger consumers tried to avoid health warnings [52, 58, 67, 86]. However, earlier studies found that there was interest in health warning labels among young consumers [50, 76, 89] along with awareness of these [53, 74, 76]. Definitions of age categories also varied across studies.

Females reported greater knowledge and interest in nutrition, calorie, and health warning labels, seeking and relying on this information for decision making [50, 64, 67, 69, 73, 76, 81, 89, 90]. They found messages believable and potentially effective in reducing alcohol intake [49, 50, 58, 64, 67, 69, 73, 76, 81, 87, 89, 90].

Health-conscious consumers used wine and food labels similarly, with higher health and alcohol awareness leading to greater interest in informative nutrition and warning labels [50–52, 63, 73, 76].

### Label Preferences

#### Content Detail

For nutrition information, preferences existed for detailed information [70, 73], though European consumers preferred simplified versions like images with kcal indications [48, 50], while United States consumers preferred detailed nutrition panels [48].

Italian consumers preferred short ingredients lists, particularly clean labelled products without ingredients [70]. One-third of consumers favoured wines with shorter ingredient lists [59].

For health warnings, Spanish and Italian consumers preferred logos with statements (full label version), while United States and French consumers preferred simple label versions with logos only [48, 50, 76].

#### Placement

Mixed results emerged for front versus back placement. When choosing wine bottles, 40% of consumers always read front labels and 26% always read back labels [50, 76]. European and United States consumers showed more interest in front nutrition information [48], while Italian consumers preferred back label placement [73].

Australian consumers preferred health warnings on back labels to reduce negative emotions and limit exposure to unpleasant messages [84]. Millennial consumers (born 1978–2000) from France and Italy preferred health warnings more for beer than wine (both on front), with no between-group comparison included in the analysis [86].

#### Design Elements

Traffic light coloured calorie labelling was preferred by some consumers, with labels incorporating a traffic light system reported to help understanding of calorie content, and some indicated that these labels would make them drink less, compared with the healthier choice tick and guideline amounts labels [58]. For pregnancy warnings, pictograms were preferred and most effective, with 97% of female consumers associating red colour with warnings [74, 91].

Consumers found colour and size (red and 50% larger) more effective in gaining health warning attention [56, 57, 75, 80, 83, 85, 92] as it was easily noticed [65, 81, 82].

#### Message Characteristics

Consumers randomised to specific warnings had higher response-efficacy compared to general warnings [58, 93]. Messages referring to specific statements like diabetes and bowel disease being linked to alcohol consumption performed better than general messages [67, 68, 89, 94–96].

Results were mixed for positive (e.g. “reduce your drinking to reduce your risk of cancer”; “drinking less reduces your risk of mental illness”; “please fill a glass properly. your alcohol moderation behaviour makes us smile with joy”) versus negative messages (e.g. “alcohol increases your risk of cancer”; “alcohol increases your risk of mental illness”; “your life insurance may have to pay out after only one glass of an alcoholic beverage”), although message framing definitions varied across studies, including the use of positive and negative reinforcement terminology [58, 71, 89]. Some studies found negative reinforcement or negatively framed messages performed slightly better [52, 58, 71, 86, 93], though differences existed across cultures [69]. Positively framed statements were more believable with personal relevance [69, 89].

Results were also mixed for pictogram versus graphic versus text messages. Pictogram/pictorial warnings were preferred over text and graphic warnings [74, 84], whereas plain package/graphic warnings were more effective in changing intentions compared to text warnings, although the effect was small [80, 97, 98]. There was awareness, recall, acceptability, believability and personal relevance for the text warnings [55, 58, 68, 69, 74, 93, 99, 100]. Text-based information was more helpful than symbols [60].

Higher preference existed for short-term consequence warnings like “do not drive after drinking” compared to long-term outcomes like maternal risk during pregnancy [50, 52, 62, 76, 85–87, 89].

## Discussion

Consumers have a complex relationship with alcohol labels. They claim limited attention and state label information is unlikely to change purchasing habits [51, 57, 64, 72]. However, food industry experience shows labels do influence purchases subconsciously [101–105]. Consumers are becoming more health-conscious, affecting food and alcohol purchases [63, 106–108].

International pressure grows to increase ingredients, nutrition and health warnings on alcohol products [109]. Australia and New Zealand require mandatory energy labelling and clarified when nutrition content claims may be made [16, 110]. The European Union implemented wine labelling requirements for energy disclosure [36]. Ireland requires calorie content and explicit health warnings [17, 20].

Consumer lack of attention, awareness, and mixed attitudes toward nutrition and ingredients labels connect to limited understanding. The findings are consistent with the broader food labelling literature, which suggests that consumers generally report confidence in their ability to use food labelling to make informed choices, although some consumers prioritise other factors such as price and experience difficulties understanding and using label information during purchasing decisions due to factors such as lack of trust, illegibility, and difficulty interpreting label content, with levels of trust and confidence varying across consumers [111, 112]. Future research should investigate making alcohol labels more understandable, particularly regarding ingredients and nutrition literacy. Evidence from food labelling research suggests that simplified interpretive formats, such as front of pack symbols or colour coded systems, may improve comprehension compared with standard nutrition information panels [113, 114]. Whether similar approaches would improve consumer understanding of alcohol labels warrants further investigation. Health warnings also lacked attention and credibility, suggesting need for further investigation of label credibility and consumer understanding.

Although this review included studies published between 2010 and 2020, more recent research has extended the evidence base. Post-2020 evidence suggests that health warnings and nutrition-related information generally improve consumer awareness, knowledge and risk perceptions, while evidence for changes in purchasing or consumption behaviour remains limited and inconsistent, suggesting a potential intention–behaviour gap between stated perceptions and behavioural outcomes [115–119]. Studies have also demonstrated that health-oriented marketing cues such as “low sugar”, “low carbohydrate” and “low calorie” may generate a health halo effect, whereby some consumers perceive certain alcoholic products as healthier or less harmful [120–122]. However, recent Food Standards Australia New Zealand consumer research found that such claims did not affect compensatory food intake or physical activity, and that consumers generally did not perceive alcoholic beverages as healthy overall [31]. At present, the prevailing regulatory position is that the population-wide benefits of transparency and consumers’ right to access nutritional information outweigh the theoretical risk of driving food or alcohol substitution, although debate continues over whether gaps in regulation may allow potentially misleading claims [123, 124]. As alcohol labelling requirements continue to evolve, these findings reinforce the importance of consumer education to support understanding and interpretation of this information.

A common conclusion was that gaining consumer attention requires multiple sources beyond labels, including public campaigns, signs, digital platforms, posters, QR codes, advertisements, and other media. This review found that those most likely to pay attention and believe information might alter consumption were females, lighter drinkers, and health-conscious consumers. This review also found mixed results regarding label placement, characteristics and contents. The food industry’s experience in developing and implementing transparent, evidence based labelling practices may provide insights for alcohol producers when building subsequent research, market, and policy considerations [125]. Further research is needed to examine optimal design, placement and content, especially for nutrition and ingredients, as most studies targeted health warnings.

## Limitations

Most articles focused on health warning labels, restricting application to ingredients and nutrition information. Articles not written in English were excluded, potentially missing themes or evidence pools for nutrition and ingredients labelling. The search was restricted to studies published between 2010 and 2020, although more recent evidence is acknowledged in the Discussion. Finally, reporting quality was assessed using the STROBE and CONSORT reporting checklists rather than formal critical appraisal tools, consistent with scoping review methodology.

## Conclusion

This review examined consumer preferences and attitudes toward alcohol labelling. Labelling information plays a significant role in consumer preferences and product acceptance.

Implementation of labelling changes is complex, with many requirements and obstacles for countries and alcohol makers. This review found positive relationships between consumers and alcohol labels, though limited. Further research is required before mandatory labelling implementation to ensure consumer protection through informed choice.

The evidence suggests that while consumers express interest in nutrition, ingredients and health warning information on alcohol labels, their actual use of this information for decision-making remains limited. Policymakers should draw upon the extensive evidence from food labelling research when developing alcohol labelling requirements, recognising that effective label design requires not only accurate information but also presentation formats that consumers can easily interpret and apply in purchasing decisions.

Successful implementation of comprehensive alcohol labelling policies will require careful consideration of consumer preferences, understanding barriers, and development of multi-channel communication strategies that extend beyond labels alone.

##  Key References

- World Health Organization. (2024). Global alcohol action plan 2022–2030. World Health Organization. https://www.who.int/teams/mental-health-and-substance-use/alcohol-drugs-and-addictive-behaviours/alcohol/our-activities/towards-and-action-plan-on-alcohol.
  - ◦This report is of outstanding importance and outlines the WHO action plan to reduce the harmful use of alcohol and supports consumer protection by emphasising the implementation of essential information about alcoholic beverages, including health warnings, ingredients, and calories, so consumers can make informed choices and understand risks.
- Alsini, N., Kutbi, H. A., Hakim, N., Mosli, R., Eid., N., & Mulla., Z. (2023). Factors influencing grocery shopping choices and the prevalence of food label use among Saudi mothers: a cross-sectional pilot study. *Nutrition and food science*,* 53* (2), 432–444. 10.1108/NFS-11-2021-0345.
  - ◦This study is of importance and outlines that consumers value food labels, and the label’s impact depends on presentation and awareness. Different demographics, such as income and education are connected to how effectively labels are used. The study also highlights the need to provide education to help consumers better understand and use food labels.
- Priya, K. M., & Alur, S. (2023). Analyzing consumer behaviour towards food and nutrition labelling: A comprehensive review. *Heliyon*,* 9* (9), e19401. 10.1016/j.heliyon.2023.e19401.
  - ◦This article is of importance and examines consumers’ perceptions of food and nutrition labels and identifies key factors that influence label use when making food choices, such as past experiences, personal beliefs, and attitudes.

## Supplementary Information

Below is the link to the electronic supplementary material.

Supplementary Material 1 (DOCX 108 KB)

Supplementary Material 2 (DOCX 37.4 KB)

Supplementary Material 3 (DOCX 50.5 KB)

Supplementary Material 4 (DOCX 27.5 KB)

Supplementary Material 5 (DOCX 77.9 KB)

Supplementary Material 6 (XLS 22.6 KB)

Supplementary Material 7 (DOC 282 KB)

## Data Availability

No datasets were generated or analysed during the current study.
