# Supplementary material for: Consumers’ Attitudes and Preferences Towards Ingredients List, Nutrition Information and Health Warning Labelling on Alcohol Products: A Scoping Review
Source: Curr Nutr Rep. 2026 Jul 22;15(1):62. doi: 10.1007/s13668-026-00784-y (PMC13391450; doi:10.1007/s13668-026-00784-y)
Supplement: Supplementary file 2 — Supplementary Material 2 (DOCX 37.4 KB) [file 13668_2026_784_MOESM2_ESM.docx]

| **Database Name** | **Database Date Range** | **Date Search** | **Complete Search Strategy** |  |  |  |
| --- | --- | --- | --- | --- | --- | --- |
| Ovid Medline | 1946-2020 | 17/12/20 | Please refer to page 2 for more information  Controlled Vocabulary used: Medical Subject Headings (MeSH) | 2010-2020 | Black | 1419 |
| Ovid Embase | 1947-2020 | 17/12/20 | Please refer to page 3 for more information  Controlled Vocabulary used: Emtree thesaurus | 2010-2020 | Black | 2617 |
| Ovid Emcare | 1995-2020 | 17/12/20 | Please refer to page 4 for more information  Controlled Vocabulary used: Emtree thesaurus | 2010-2020 | Black | 662 |
| EBSCohost Business Source Complete | 2010-2020 | 23/12/20 | Please refer to pages 5-8 for more information | 2010-2020 | Black | 589 |
| ABI/  INFORM collection  ProQuest | 2010-2020 | 14/12/20 | “Consumer” AND “alcohol” AND (“nutrition” OR “health warning” OR “ingredient”) | 2010-2020 | Black | 50 |
| Emerald Insight | 2010-2020 | 14/12/20 | “Consumer” AND “alcohol label” AND (“nutrition” OR “health warning” OR “ingredient”) | 2010-2020 | Black | 395 |
| Business collection  Informit | Advanced search | 15/12/20 | (alcohol label) AND consumer OR (health warning) OR ingredient OR nutrition | 2010-2020 | Black | 68 |
| Web of Science | Basic search Databases:  WOS, CCC, KJD, MEDLINE, RSCI, SCIELO. | 23/12/20 | Please refer to page 9 for more information | 2010-2020 | Black think | 78 |
| Scopus | 2010-2020 | 23/12/20 | Please refer to page 9 for more information |  | Grey | 2000 |
| ProQuest central | Advanced Search | 18/12/20 | Please refer to page 9 for more information | 2010-2020 | Grey | 2092 |
| ProQuest dissertation and theses | Advanced search | 23/12/20 | Please refer to page 9 for more information | 2010-2020 | Grey | 390 |
| Google Scholar | Access via UniSA | 15/12/20 | “alcohol labelling” AND consumer AND nutrition AND health warning AND ingredient | 2010-2020 | Grey | 83 |

**Appendix 2: Search strategies used in the scoping review (this appendix includes the databases searched, detailed search terms and date ranges)**

Database(s): **Ovid MEDLINE**(R) ALL 1946 to December 17, 2020
Search Strategy:

| # | Searches | Results |
| --- | --- | --- |
| 1 | Consumer Behavior/ | 21891 |
| 2 | Health Behavior/ | 51224 |
| 3 | Health Knowledge, Attitudes, Practice/ | 114171 |
| 4 | Attitude/ | 47893 |
| 5 | Habits/ | 4957 |
| 6 | Decision Making/ | 96951 |
| 7 | Choice Behavior/ | 32943 |
| 8 | Food Preferences/ | 14656 |
| 9 | (preference* or behav* or attitude* or opinion* or observation* or knowledge* or aware* or recogni* or decide* or decision* or habit* or choice* or choos* or select* or pick* or like or likes or liking or liked).tw,kf. | 6846209 |
| 10 | or/1-9 | 6951875 |
| 11 | Product Labeling/ | 2650 |
| 12 | Food Labeling/ | 3866 |
| 13 | (label* or packag* or health warning* or (content* adj3 (sticker* or tag* or symbol* or ticket*)) or ((warning* or ingredient* or nutrition* or energy or kilojoule* or calorie*) adj5 (list* or declar* or table or statement*))).tw,kf. | 625859 |
| 14 | or/11-13 | 628463 |
| 15 | 10 and 14 | 189837 |
| 16 | exp Alcoholic Beverages/ | 20505 |
| 17 | Alcohol Drinking/ | 68003 |
| 18 | Beer/ | 3497 |
| 19 | Wine/ | 10918 |
| 20 | (alcohol* or spirits or beer* or wine* or liqueur*).tw,kf. | 371424 |
| 21 | or/16-20 | 387366 |
| 22 | 15 and 21 | 2409 |
| 23 | limit 22 to yr="2010 - 2020" | 1419 |

Database(s): **Embase** Classic+Embase 1947 to December 17, 2020
Search Strategy:

| # | Searches | Results |
| --- | --- | --- |
| 1 | consumer attitude/ | 5276 |
| 2 | health behavior/ | 68757 |
| 3 | attitude to health/ | 118054 |
| 4 | attitude/ | 72023 |
| 5 | habit/ | 26052 |
| 6 | decision making/ | 242884 |
| 7 | food preference/ | 14269 |
| 8 | (preference* or behav* or attitude* or opinion* or observation* or knowledge* or aware* or recogni* or decide* or decision* or habit* or choice* or choos* or select* or pick* or like or likes or liking or liked).tw,kw. | 9010616 |
| 9 | or/1-8 | 9175789 |
| 10 | packaging/ | 8182 |
| 11 | food packaging/ | 10877 |
| 12 | (label* or packag* or health warning* or (content* adj3 (sticker* or tag* or symbol* or ticket*)) or ((warning* or ingredient* or nutrition* or energy or kilojoule* or calorie*) adj5 (list* or declar* or table or statement*))).tw,kw. | 839050 |
| 13 | or/10-12 | 845303 |
| 14 | 9 and 13 | 262295 |
| 15 | exp Alcoholic Beverages/ | 33712 |
| 16 | drinking behavior/ | 51940 |
| 17 | beer/ | 8188 |
| 18 | wine industry/ or wine/ | 15525 |
| 19 | (alcohol* or spirits or beer* or wine* or liqueur*).tw,kw. | 535636 |
| 20 | or/15-19 | 555272 |
| 21 | 14 and 20 | 3925 |
| 22 | limit 21 to yr="2010 - 2020" | 2634 |

Database(s): **Ovid** **Emcare** 1995 to December 17, 2020
Search Strategy:

| # | Searches | Results |
| --- | --- | --- |
| 1 | consumer attitude/ | 2021 |
| 2 | health behavior/ | 35347 |
| 3 | attitude to health/ | 16451 |
| 4 | attitude/ | 10951 |
| 5 | habit/ | 14552 |
| 6 | decision making/ | 99882 |
| 7 | food preference/ | 4498 |
| 8 | (preference* or behav* or attitude* or opinion* or observation* or knowledge* or aware* or recogni* or decide* or decision* or habit* or choice* or choos* or select* or pick* or like or likes or liking or liked).ab,ti,kw. | 1892410 |
| 9 | or/1-8 | 1934807 |
| 10 | packaging/ | 2772 |
| 11 | food packaging/ | 2607 |
| 12 | (label* or packag* or health warning* or (content* adj3 (sticker* or tag* or symbol* or ticket*)) or ((warning* or ingredient* or nutrition* or energy or kilojoule* or calorie*) adj5 (list* or declar* or table or statement*))).ab,ti,kw. | 101449 |
| 13 | or/10-12 | 103106 |
| 14 | 9 and 13 | 38220 |
| 15 | exp Alcoholic Beverages/ | 9540 |
| 16 | drinking behavior/ | 16752 |
| 17 | beer/ | 2569 |
| 18 | wine industry/ or wine/ | 3501 |
| 19 | (alcohol* or spirits or beer* or wine* or liqueur*).ab,ti,kw. | 119191 |
| 20 | or/15-19 | 123977 |
| 21 | 14 and 20 | 953 |
| 22 | limit 21 to yr="2010 - 2020" | 668 |

**EBSCohost Business Source** **Complete** 23 December 2020

accessibility Information and TipsRevised Date: 07/2015

Print Search History

| 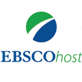 | |  | | | |
| --- | --- | --- | --- | --- | --- |
| # | Query | | Limiters/Expanders | Last Run Via | Results |
| S9 | (SU ( alcohol* or spirits or beer* or wine* or liqueur* ) OR TI ( alcohol* or spirits or beer* or wine* or liqueur* ) OR AB ( alcohol* or spirits or beer* or wine* or liqueur* ) OR KW ( alcohol* or spirits or beer* or wine* or liqueur* )) AND (S5 AND S6 AND S7) | | Limiters - Published Date: 20100101-20211231 Search modes - Boolean/Phrase | Interface - Research Databases Search Screen - Advanced Search Database - Business Source Complete | 665 |
| S8 | (SU ( alcohol* or spirits or beer* or wine* or liqueur* ) OR TI ( alcohol* or spirits or beer* or wine* or liqueur* ) OR AB ( alcohol* or spirits or beer* or wine* or liqueur* ) OR KW ( alcohol* or spirits or beer* or wine* or liqueur* )) AND (S5 AND S6 AND S7) | | Search modes - Boolean/Phrase | Interface - Research Databases Search Screen - Advanced Search Database - Business Source Complete | 1,445 |
| S7 | SU ( alcohol* or spirits or beer* or wine* or liqueur* ) OR TI ( alcohol* or spirits or beer* or wine* or liqueur* ) OR AB ( alcohol* or spirits or beer* or wine* or liqueur* ) OR KW ( alcohol* or spirits or beer* or wine* or liqueur* ) | | Search modes - Boolean/Phrase | Interface - Research Databases Search Screen - Advanced Search Database - Business Source Complete | 201,784 |
| S6 | SU ( (label* or packag* or "health warning*" or (content* n3 (sticker* or tag* or symbol* or ticket*)) or ((warning* or ingredient* or nutrition* or energy or kilojoule* or calorie*) n5 (list* or declar* or table or statement*))) ) OR TI ( (label* or packag* or "health warning*" or (content* n3 (sticker* or tag* or symbol* or ticket*)) or ((warning* or ingredient* or nutrition* or energy or kilojoule* or calorie*) n5 (list* or declar* or table or statement*))) ) OR AB ( (label* or packag* or "health warning*" or (content* n3 (sticker* or tag* or symbol* or ticket*)) or ((warning* or ingredient* or nutrition* or energy or kilojoule* or calorie*) n5 (list* or declar* or table or statement*))) ) OR KW ( (label* or packag* or "health warning*" or (content* n3 (sticker* or tag* or symbol* or ticket*)) or ((warning* or ingredient* or nutrition* or energy or kilojoule* or calorie*) n5 (list* or declar* or table or statement*))) ) | | Search modes - Boolean/Phrase | Interface - Research Databases Search Screen - Advanced Search Database - Business Source Complete | 364,342 |
| S5 | SU ( preference* or behav* or attitude* or opinion* or observation* or knowledge* or aware* or recogni* or decide* or decision* or habit* or choice* or choos* or select* or pick* or like or likes or liking or liked ) OR TI ( preference* or behav* or attitude* or opinion* or observation* or knowledge* or aware* or recogni* or decide* or decision* or habit* or choice* or choos* or select* or pick* or like or likes or liking or liked ) OR AB ( preference* or behav* or attitude* or opinion* or observation* or knowledge* or aware* or recogni* or decide* or decision* or habit* or choice* or choos* or select* or pick* or like or likes or liking or liked ) OR KW ( preference* or behav* or attitude* or opinion* or observation* or knowledge* or aware* or recogni* or decide* or decision* or habit* or choice* or choos* or select* or pick* or like or likes or liking or liked ) | | Search modes - Boolean/Phrase | Interface - Research Databases Search Screen - Advanced Search Database - Business Source Complete | 2,939,873 |
| S4 | S1 AND S2 AND S3 | | Search modes - Boolean/Phrase | Interface - Research Databases Search Screen - Advanced Search Database - Business Source Complete | 1,283 |
| S3 | SU ( wine OR beer OR alcohol ) OR TI ( alcohol* or spirits or beer* or wine* or liqueur* ) OR AB ( alcohol* or spirits or beer* or wine* or liqueur* ) OR KW ( alcohol* or spirits or beer* or wine* or liqueur* ) | | Search modes - Boolean/Phrase | Interface - Research Databases Search Screen - Advanced Search Database - Business Source Complete | 194,786 |
| S2 | SU ( labels OR "packaging labels" OR "food labeling" ) OR TI ( (label* or packag* or "health warning*" or (content* n3 (sticker* or tag* or symbol* or ticket*)) or ((warning* or ingredient* or nutrition* or energy or kilojoule* or calorie*) n5 (list* or declar* or table or statement*))) ) OR AB ( (label* or packag* or "health warning*" or (content* n3 (sticker* or tag* or symbol* or ticket*)) or ((warning* or ingredient* or nutrition* or energy or kilojoule* or calorie*) n5 (list* or declar* or table or statement*))) ) OR KW ( (label* or packag* or "health warning*" or (content* n3 (sticker* or tag* or symbol* or ticket*)) or ((warning* or ingredient* or nutrition* or energy or kilojoule* or calorie*) n5 (list* or declar* or table or statement*))) ) | | Search modes - Boolean/Phrase | Interface - Research Databases Search Screen - Advanced Search Database - Business Source Complete | 345,801 |
| S1 | SU "CONSUMER attitudes" OR TI ( preference* or behav* or attitude* or opinion* or observation* or knowledge* or aware* or recogni* or decide* or decision* or habit* or choice* or choos* or select* or pick* or like or likes or liking or liked ) OR AB ( preference* or behav* or attitude* or opinion* or observation* or knowledge* or aware* or recogni* or decide* or decision* or habit* or choice* or choos* or select* or pick* or like or likes or liking or liked ) OR KW ( preference* or behav* or attitude* or opinion* or observation* or knowledge* or aware* or recogni* or decide* or decision* or habit* or choice* or choos* or select* or pick* or like or likes or liking or liked ) | | Search modes - Boolean/Phrase | Interface - Research Databases Search Screen - Advanced Search Database - Business Source Complete | 2,723,396 |

Bottom of Form

**Web of Science**

(preference* or behav* or attitude* or opinion* or observation* or knowledge* or aware* or recogni* or decide* or decision* or habit* or choice* or choos* or select* or pick* or like or likes or liking or liked) AND TOPIC: (label* or packag* or "health warning*" or (content* near/3 (sticker* or tag* or symbol* or ticket*) ) or ((warning* or ingredient* or nutrition* or energy or kilojoule* or calorie*) near/5 (list* or declar* or table or statement*) )) AND TOPIC: (alcohol* or spirits or beer* or wine* or liqueur*)

Indexes=SCI-EXPANDED, SSCI, A&HCI, CPCI-S, CPCI-SSH, ESCI Timespan=2010-2020

**Scopus**

( TITLE-ABS-KEY ( ( consumer*  AND preference*  OR  behav*  OR  attitude*  OR  opinion*  OR  observation*  OR  knowledge*  OR  aware*  OR  recogni*  OR  decide*  OR  decision*  OR  habit*  OR  choice*  OR  choos*  OR  select*  OR  pick*  OR  like  OR  likes  OR  liking  OR  liked ) )  AND  TITLE-ABS-KEY ( ( label*  OR  packag*  OR  "health warning*"  OR  ( content*  W/3  ( sticker*  OR  tag*  OR  symbol*  OR  ticket* ) )  OR  ( ( warning*  OR  ingredient*  OR  nutrition*  OR  energy  OR  kilojoule*  OR  calorie* )  W/5  ( list*  OR  declar*  OR  table  OR  statement* ) ) ) )  AND  TITLE-ABS-KEY ( ( alcohol*  OR  spirits  OR  beer*  OR  wine*  OR  liqueur* ) ) )  AND  PUBYEAR  >  2009  AND  ( LIMIT-TO ( LANGUAGE ,  "English" ) )

**ProQuest Central**

(noft(consumer* preference* OR behav* OR attitude* OR opinion* OR observation* OR knowledge* OR aware* OR recogni* OR decide* OR decision* OR habit* OR choice* OR choos* OR select* OR pick* OR like OR likes OR liking OR liked) AND noft(alcohol (label* OR packag* OR "health warning*" OR (content* NEAR/3 (sticker* OR tag* OR symbol* OR ticket*)) OR ((warning* OR ingredient* OR nutrition* OR energy OR kilojoule* OR calorie*) NEAR/5 (list* OR declar* OR table OR statement*))))) AND noft(alcohol* OR spirits OR beer* OR wine* OR liqueur*)

No variations English only excludes duplicates

**ProQuest dissertation and theses**

noft(preference* OR behav* OR attitude* OR opinion* OR observation* OR knowledge* OR aware* OR recogni* OR decide* OR decision* OR habit* OR choice* OR choos* OR select* OR pick* OR like OR likes OR liking OR liked) AND noft((label* OR packag* OR "health warning*" OR (content* NEAR/3 (sticker* OR tag* OR symbol* OR ticket*)) OR ((warning* OR ingredient* OR nutrition* OR energy OR kilojoule* OR calorie*) NEAR/5 (list* OR declar* OR table OR statement*)))) AND noft(alcohol* OR spirits OR beer* OR wine* OR liqueur*)
