# Supplementary material for: Consumers’ Attitudes and Preferences Towards Ingredients List, Nutrition Information and Health Warning Labelling on Alcohol Products: A Scoping Review
Source: Curr Nutr Rep. 2026 Jul 22;15(1):62. doi: 10.1007/s13668-026-00784-y (PMC13391450; doi:10.1007/s13668-026-00784-y)
Supplement: Supplementary file 3 — Supplementary Material 3 (DOCX 50.5 KB) [file 13668_2026_784_MOESM3_ESM.docx]

**Appendix 3: Table I Summary of articles included in review that examine the nutrition information**

| **Author/ Year** | **Country** | **Type of Alcohol** | **Study design/ method according to the study authors** | **Population Sample** | **Outcome/ Comments** |
| --- | --- | --- | --- | --- | --- |
| Annunziata et al. 2016a | Italy, France, Spain, United States of America | Wine | Quantitative Research; Cross-country online survey; conjoint design. | n=1016  330 in Italy;  185 in France;  195 in Spain;  306 in USA (east coast)  51% female  European respondents 18-70 years old, drinking wine at least once a month; USA respondents 21-70 years old, drinking wine at least once a month | Aim: Analysed respondents’ behaviour, label use and belief, knowledge, and interest in nutrition wine labels information, and their preferences for additional information.  Results: Respondents had difficulty understanding the nutrition label information and calorie content of wine. Europeans prefer a simplified version (e.g., an image with an indication of calories), whereas the USA respondents prefer a detailed nutritional panel. 15% of European’s compared to 24% of USA respondents, stated that they changed their habits after reading nutrition labels, while 20% of European and 40% of USA respondents reported they would not change. Respondents' attitudes towards nutritional information are also related to consumption habits, the degree of connection with wine, and socio-demographic elements or geographic origin. Respondents preferred information on the front label rather than the back. Respondents were segmented into four groups. Group 1 (22% of the sample, preferred nutrition information, had more knowledge of it, paid more attention, and were more interested in allergenic substances. They were mainly females between 35-55 years, had a higher level of education, and more USA respondents); Group 2 (35% of the sample, read the nutrition information when shopping and found it too hard to understand. They were mostly females below age 45); Group 3 (28% of the sample, dedicate minimal time when reading nutrition labels, overestimate the calorie content, mostly males over 35 with average educational level); Group 4 (15% of sample “poor attitude” towards nutrition labels as mostly never read them, knew little about calorie content, have more frequent consumption of wine). Although there is a difference in clusters overall and between countries, respondents have an interest in and support nutrition information on wine labels. Implementation costs should be taken into consideration. |
| Annunziata et al.  2016c | Italy | Wine | Quantitative Research; Questionnaire Survey; conjoint design. | n=300 Italy:  28% Campania,  27% Emilia Romagna,  25% Lombardy,  20% Lazio  18-75 years  51% female | Aim: Analysed respondents’ preferences and interest in nutrition and health warnings on wine labelling.  Results: 48% were poorly informed about the nutritional properties of wine, 12% were very well informed. 55% reported it would be useful to receive more nutrition characteristics information of wine through the label. 27% wanted and are interested in nutritional information found in the form of the wine glass with kcal on. 20% were correct in kcal identification. 29% disagreed that wine contributes to obesity. They were least interested in allergens. Believe that alcohol, particularly wine, can have beneficial effects on health if consumed in moderation. There were 3 different consumer segments that cluster analysis identified: (1) 25% of respondents were detailed information seekers; these were mainly female consumers (over 55) assigning a high utility for nutrition and preferred the full version of nutritional information label (e.g. panel with GDA%), pay attention to the back label and were able to identify calories content; (2) 48% of respondents were health warning seekers mainly younger men (under 44) and had a high interest in health warnings and low knowledge of nutrition labels, they preferred a picture card with the glass indicating the amount of calorie, pay less attention to label information (3) (aged 45-54) 27% of responders were classed as simplified information seekers with a relatively low educational level, and who assign relatively high importance to nutrition information, they preferred product together with glass that indicates the kcal amount, had difficulty in interpretation of nutrition. Recommends the implementation of nutritional advice in the form of a glass with calorie content on it, as this is cheaper and easier to implement. |
| Bazzani et al. 2019 | Italy | Red wine | Quantitative Research; Online survey incorporating a choice experiment design. | n=278  18-74 years  52.52% female  Drinking red wine more than once a month | Aim: Explored consumer use of information currently on wine labels and their preferences for information around ‘naturalness’ such as clean labels and alcohol content.  Results: There was low knowledge about the link between nutrition compounds and wine. There was an interest in using labels to choose wine and food products by those who paid attention to their health and weight. Those with wine interests and health-oriented read wine labels more. Nutrition knowledge was connected to food label use but not wine. Attitudes towards reading and using wine labels, as in the instance of food labels, increased in health consciousness-orientated respondents. There was a majority preference for nutrition information such as clean labels (organic and natural) and lower alcohol content, especially for health-conscious and younger respondents. The paper identified that wine consumers appreciated having nutritional information on labels compared to other alcoholic beverage consumers. It could also be an incentive to moderate use in the home environment. |
| Clarke 2017 | United Kingdom | Study 5 Beer  Study 6  Glass stimuli were developed via a google image search of common drinks (glass of  wine, pint of beer, gin and tonic, double rum and coke, jagerbomb) | PhD thesis with multiple studies.  Study 5: Between subjects design; Questionnaires; Quantitative and  Qualitative questions.  Study 6: Between subjects design with random assigning.  Study 7: Qualitative, 2 Focus Groups. | Study 5  n=100  Weekly consumption of alcohol (social drinkers) (52 female; mean age 22.4)  Study 6  n=142 (107 female; 23.79 years )  Study 7  n=14 social drinkers (8 females; mean age 23) | Multiple studies but only three relate to the question.  Study 5 Aim: Labelled glasses were explored, and their effects on ad libitum alcohol consumption.  Results: There were 79% risky drinkers. Males consumed more than females. Effects on consumption reduction only in females when it came to exercise labels compared to unit and calorie labels. When it came to views and beliefs of the labels and their influences on alcohol consumption, respondents' responses were neutral 91%, 46% were of belief there is no influence on consumption or their behaviour from labels, and 22% thought there is. 17% did not believe the labels influenced their consumption but did increase their awareness. Overall, glass labels were not effective in changing alcohol consumption behaviour.  Study 6 Aim: Explored the labelling effects on drinking behavioural intentions.  Results: There were 42% risky drinkers. Regarding respondents’ number of drinks they planned to consume in the upcoming week, there were no differences between four labelled glass conditions and their likelihood of becoming drunk the following week. The introduction on labels on the glass was not associated with a reduction in respondents’ intention to consume alcohol. When asked to recall the information respondents had seen, the recall was highest for the volume label at 95%, then the exercise label at 88%, the food label at 85%, and 80% for the calories/unit label. Overall, labels on the glass were not effective in changing alcohol-drinking intentions. Studies 5 and 6 concluded that labels were ineffective in changing drinking behaviour overall.  Study 7 Aim: Views of social drinkers - respondents were investigated on labelled glasses to potentially reduce alcohol consumption (continuation from study 6).  Results: 92.86% were risky drinkers. Respondents viewed all labels as positive, noticeable (due to being novel, particularly exercise, and food labels), and beneficial (due to giving them as much information as possible). Mostly females thought that their behaviour would be changed with the labels and they would reduce consumption, and labels displaying food and exercise were most effective to them because they had more impact and were more relatable. Females also implied that drinks with lower calories might be wanted and higher alcohol content instead. Males believed that labels would not affect behaviour change; they would not count the calories, and even when they would count, they would ignore calories; they have found exercise labels most effective. Males thought labels could be useful for self-monitoring purposes. Some respondents were surprised to learn about the drinks' calorie numbers. Respondents thought that labels could be used negatively as food restriction before drinking, they may lead to an increase in consumption, used as a counterproductive, harmful approach- leading to compensatory exercise or eating behaviours, drinking games, would change burn off calories dynamics and supplementary measures would be needed to prevent this information being misused. They believed that labels would not be useful for themselves but for those who want to help themselves rather than for their benefit, who want to cut down consumption, and who are weight conscious. Others stated that labels were not noticeable, upsetting, or bad for the industry and that they would not pay attention and would become habituated to them. Also, once in the drinking environment, the labels will be less likely to lead to or impact a behaviour change. Overall, glass labels were not effective in changing behaviour. However, respondents thought glass calorie labels were useful and noticeable and may lead to changing intentions. |
| Escandon-Barbosa and Rialp-Criado 2019 | Colombia | Wine | Observational study; Hierarchical model methodology. | n=114  (32% female  67% male)  25-54 years old  Consuming at least one glass of wine per week | Aim: Analysed the contents of labels on respondents' (consumption expert/non-expert and gender) buying intentions (non-expert: low and less frequent consumption, expert: higher and more frequent).  Results: Information needs and attitudes towards labelling can be affected by gender, as information is processed differently. Label information is highly relevant to respondents, they read information differently, and this does impact their buying intention. Comparing experts to non-experts and females to males, nutrition information alone, compared to being combined with other label parts, have positive influences on their purchase intention. There was a greater effect on experts than on non-experts. Three components of the label (denomination of origin, health warnings, and nutritional information) were considered by the experts, increasing their intention to purchase. Non-experts reported that these were confusing, and these components were not used to determine their intent to purchase. Females placed greater emphasis on nutrition information for their decision-making, either on its own or with the other two components. Males were less likely to read labels and relied less on this information. |
| GFK. Consumer Insights Report 2016 | Germany, Poland, Denmark, Netherlands, Spain, United Kingdom, Italy, France Romania | Beer, wine, spirits | Quantitative Research; Online survey with random sampling. | n=9,008  18- 65 years old  Majority female  Denmark n=1001;  Poland n=1001;  Spain n=1001;  Netherlands n=1002;  Germany n=1001;  United Kingdom n=1001;  France n=1002;  Romania n=1000;  Italy n=1000 | Aim: Examined consumers’ call for all alcoholic beverages to receive the same ingredients and the full nutrition information per 100ml as for other products.  Results: 86% of respondents believe they should have access to the same nutrition information for alcoholic beverages (wine, beer, spirits) as per 100ml as they currently receive for any other drink and food product, up from 69% in 2014. 71% were interested to see the energy and full nutrition information. However, there are cultural influences; Italy showed the highest interest, 91% energy value, and 91% full nutrition information. The Netherlands showed the lowest 48% energy value and 49% full nutrition information. The authors found a pattern between the frequency of alcohol consumption and interest in the information; regular drinkers or those who consumed more were less interested in the information. About a 3rd only would focus solely on one information source, whilst more than half would use two or more. Almost half would use a mix of digital online sources (such as applications and websites) and traditional information sources (such as labels, advertising, and in-store communication). For example, to access nutrition information a majority of respondents would use a variety of sources; nearly 2/3 are considering two or more sources. 44% used a mixture of digital online and traditional offline sources, 31% would use traditional and 17% digital. 74% would use brand-supported information sources to access nutrition information, such as brand labels or websites. When observing the label used as one of the information sources, 71% would use it (still the most popular but not the only source of information) as a source to access the information. When observing the label used as one as the only source to access the information, 21% would use the label. 57% are considering more than one information source to access nutrition information. |
| Grunert, Hieke and Juhl 2018 | Six European  Countries: Denmark, Germany, Netherlands, Poland, Spain,  United Kingdom | Beer, wine, gin, vodka,  whiskey and rum | Quantitative Research; Online survey. | n=5395  18-65 years  Denmark n=787 (51% male);  Germany n=993 (51% male); Netherlands n=934 (54% female);  Poland n=1003 (51% male);  Spain n=810 (56% male);  United Kingdom n=868 (54% female) | Aim: Examined to what extent ingredient and nutrition information from off-label sources is wanted and used by respondents, how this is affected by health interest, involvement with the product and previous knowledge of this information.  Results: Respondents’ had a medium level of interest in nutrition information and low levels when it came to information use. Spain had the highest nutrition information interest and a general interest in a product across all sources, and Denmark had the lowest. Spain also had the highest nutrition information usage across all sources, and Denmark and Netherlands had the lowest. There are also cultural differences. Most interest is in neutral sources, for example, getting information from public and health websites and stores, and the least is in apps and advertising. In-store information was highest for information use (self-reported). Highest nutrient knowledge for wine, relatively low for beer, and lowest for whiskey. For older respondents, the nutrition knowledge was lower. Males are more interested in the product, whereas females have more health interest, and it becomes stronger with age. The study suggested that the differences among respondents were not based on the source of information but were related more to the wants and use of information instead, such as general interest. The levels of knowledge among the countries had the same differences in information wants and use. Respondents' want and use of nutrition information were driven by having a general interest in the product more than for health reasons. Motivation for information was still there for those who are health conscious, as it had an effect on information wants. General interest in a product and its knowledge seemed to be the most important influence on information wants and use. |
| Martinez et al. 2015 | United States of America | Beer (Study 1)  Beer (Study 2)  Beer, wine, vodka (Study 3) | Between-subjects experiment in which participants were randomly allocated; Quantitative design; online survey.  Study 1 experimental study;  Study 2 extended and improved experimental study- online experiment;  Study 3 survey methodology. | Study 1  n=80 respondents underage college drinkers (75.5% female) mean age 18.56  Study 2  n=98 respondents community  drinkers (42.6% female and averaged 26.53 years)  Study 3  n=191 community drinkers  18+(59.2% female and averaged 36.49 years) | Aim: Examined how nutrition labels affect drinking consumption and impact respondents’ drinking plans and beliefs.  Results: Study 1. Nutrition labels had no effect on respondents’ drinking plans, as 36.3% stated they would still drink heavily twice per week or more. Their health and alcohol consumption beliefs were not changed by the nutrition labels’ influence. Study 2. This group of respondents was less heavy drinkers, although 7.5% stated they would still heavily drink twice per week or more. Nutrition labels had minimal effect on their planned alcohol consumption and alcohol-related beliefs. Only the type of label had a small effect on respondents’ beliefs as an accurate label made the respondents feel peaceful as opposed to the falsely reduced/extreme low-calorie label. Study 3. Their alcohol consumption beliefs were not changed by the nutrition labels’ influence. It seemed that their daily drinking plans were influenced by alcohol preference rather than information type preference. There is a difference when it comes to the respondent’s preferences regarding the type of alcohol label information, as they preferred those labels with fewer/low/decreased calories, whilst their preferences were similar for no information and accurate labels presented, especially for beer and vodka. 83% felt that their consumption would not be affected by the nutrition labels. They also expressed the benefits of the labels, such as informing them about the calories, their consumption, and buying, as well as assisting them in making healthy choices. They expressed that the downside was that they could lead to a false belief that there is some benefit in alcohol consumption. Overall, the results showed that nutritional labels did not affect drinking habits and beliefs, but respondents preferred to have low-calorie alcoholic products. While these studies demonstrated no effect of nutritional labels on alcohol consumption, 86.1% to 87% of respondents preferred having nutritional information on the label. |
| Maynard et al. 2018a | United Kingdom | Beer/cider, wine, spirits, alcopops | Quantitative design; Experimental study; Between subject design; Online survey. | n=450 respondents  18+  female 54%  male 46%  median age 34 | Aim: Investigated attitudes, knowledge, intentions, beliefs, and behaviours impacted by units, calories, and health warning alcohol labels. Also looked at the labels’ best information, delivery, and presentation.  Results: High level of support as respondents agree that calorie (81%) and unit (91%) information were a good idea and for the increased information on alcoholic beverages. 83% of the harmful drinking group supported calorie labelling. When it comes to knowledge, respondents overestimated calories. 90% said that traffic lights and traffic lights/guidelines amount labels were helpful in their calorie understanding. 22% indicated that the traffic light with guidelines amounts label would make them consume alcohol less. Followed by guidelines only label 19%, and traffic lights only label 17%. When it comes to their beliefs and attitudes, respondents preferred traffic lights with guidelines amount label 63%. After being provided with personalised calorie labels, 83% highly agreed with the inclusion of these on alcoholic beverages. However, looking at behaviour and intentions, a third of respondents (38%) indicated that they would not change their consumption, and 16% stated that these labels would influence their drinking reduction. 15% stated these calorie labels would reduce their food consumption before consuming alcohol, and 37% find their diet important. Calorie labels influence on alcohol reduction was higher for females, 19%, than males, 13%. Compared to females and lighter drinkers, heavier drinkers, and males were the ones to choose stronger alcohol drinks and get drunk. Overall, calorie labels had a positive effect on respondents’ consideration of consumption behaviour. |
| Maynard et al. 2018b | United Kingdom | Beer | Qualitative component; Experimental study; Between subjects design;  Experimental human laboratory study, ad libitum taste test. | n=264  18+ years  Drinking at least two units per week | Aim: In the ad libitum taste test, the calorie and unit information impact was investigated on the consumption behaviour of respondents.  Results: Both calorie and unit information showed no impact on consumption and intentions of alcohol in the future and on drink enjoyment, alcohol cravings, and respondent's mood. Drink enjoyment was slightly reduced with calories, but not units, and some evidence stated that those assigned to labels that could recall calories drank more than those assigned to no information. Respondents were unable to recall the percentage of calories and units in their drinks as it was low, and they were unaware of calories in their beverages. Getting drunk was the respondent's primary motivation for drinking, not thinking about calories. It was believed that calorie information might encourage healthier drinking for others and those weight conscious but not themselves and would not affect their drinking habits. Females were more likely to consider calorie information when consuming alcohol. There were also negative consequences as some respondents would reduce food consumption prior to alcohol drinking instead of alcohol reduction and would be swapping units with calories. |
| Moore 2010 | United States of America | Beer, wine distilled spirits | Quantitative and qualitative research; Survey 1: Opinion Poll survey;  Survey 2: National randomised  Survey;  Survey 3: Follow-up telephone Survey. | Survey 1  n=1,042  18+ years  Survey 2  503n respondents  18+ years  Survey 3  n=1,003  21+ years | Aim: Survey 1: Opinion poll to explore consumer support for change in labelling information.  Results: Support for information about the alcohol content (93%) as it would help respondents with better consumption and purchasing decisions. Main findings related to ingredients information.  Aim: Survey 2: Determined what type of labelling information respondents think is helpful.  Results: 90% of respondents supported mandatory labelling on all wine, spirits, and beer beverages. 79% agreed that these need to include nutrition and ingredient information. Respondents identified their priorities for information on labels; alcohol amount 92%, calories 84%, carbohydrates 75%, fat 71%, and protein 66%. Respondents supported the use of labels as it would provide education about drinking in moderation as outlined in the dietary guidelines. 60% of respondents were familiar with the guidelines, while 79% stated that it would be helpful for the guidelines to outline moderate consumption levels for females (up to 1 drink per day) and males (up to 2 drinks per day). 76% prefer the complete label that includes carbohydrates, calories, protein, and fat, as well as alcohol per serving amount and the standard drink statement.  Aim: Survey 3: Alcohol label information that respondents consider most important.  Results: 77% of respondents preferred alcohol content, 73% alcohol per serving amount, 65% calories, 57% carbohydrates, 57% can or bottle servings, 52% fat, and 46% protein. Outlined overall that respondents are in favour of complete label information as it is needed to allow ingredients about product formulations, calories for weight control, moderate drinking advice, and product comparing information. |
| Pabst et al. 2021 | Australia  Germany  Italy | Wine | Quantitative Research; Discrete choice experiment; Random selection; between and within subjects. | n=745n Australia (51% male)  n=716 Germany (57% female)  n=715 Italy (52% female)  Had to drink wine at least once a month | Aim: Examined labelling of nutrition and ingredients on wine choices of respondents and their reactions toward labelling.  Results: Respondents from all three countries were in favour of detailed nutrition information on wine and indicated it would influence their choices. They preferred detailed nutrition information, similar to other foods, such as sugar, energy, carbohydrates, salt, fats, protein, and saturated fatty acids. When making their purchasing wine decisions, high attributes/concept importance was given to nutrition back label information. In Italy, nutrition information only came second after region. Although it was lower, there was also attributes/concept importance placed to the nutrition information in Australia and Germany of 10%. Sensory descriptions in Australia and Germany when choosing wine were found to be more important (of 50%), followed by price. There were differences in respondents' reactions from new world wine markets, that is, Australia, and old/traditional world wine markets, that is, Germany and Italy, which reaction was strong towards nutrition labelling; however, across all three countries, respondents had a high preference for detailed and long nutrition information. |
| Pabst, Szolnoki and Mueller Loose 2019 | Germany | Wine | Qualitative Research; Focus groups; Three focus groups and observation of choices. | n=21  11 male  Had to drink wine at least twice a month but not be seen as wine experts | Aim: Examined respondent's reaction to nutrition and ingredient labelling: how important it is, whether there is an influence on wine demand, and the effect on respondents' attitudes to wine being a natural product.  Results: When observing the nutrition label information for the first time, there was confusion, insecurity, and incomprehension among respondents. Only 1/3 of respondents who looked at the label presented on the back of the bottle noticed new nutrition information. Respondents had little knowledge of the nutritional information of wine and did not review energy values. 76% of respondents overestimated the energy/calories of wine. All respondents agreed that energy labelling would not reduce the consumption of wine, and they do not care about it because wine is considered to be a special treat and it is only useful for those with health problems or weight concerns. Allergy labelling was supported, especially for those in need. Respondents did not support nutrition information as it was not useful and was not supported by half of the respondents. They were not interested and believed it was unnecessary and did not add any value to the product. It was even stated that it may lead to greater wine consumption. They prefer insightful information like a winery or tasting notes. Only 14% of respondents supported nutrition labelling for legal reasons and uniformity with food labelling. In the buying decision, the back label plays a minor part, and other aspects, like price, bottle design, occasion, or recommendations, are more important. All respondents agreed that energy labelling would not influence wine consumption and that nutritional labels would not change their drinking behaviour. |
| Roderique-Davies et al. 2020 | United Kingdom | Wine, cider, beer, spirits and sparkling wines | Mixed methods; Observational study; qualitative.  Qualitative Research; Focus groups  1st study eye-tracker device;  2nd study, 3 focus groups. | Study 1  25n respondents  (14 female-11 male)  23-63;  18+  Consumers of alcohol  Study 2  10n respondents  (8 females-2 males) mean age 33.9;  18+  Consumers of alcohol drinking | Aim: Investigated what label aspects respondents attend to while purchasing alcohol products, including health messages. The studies mainly focussed on health warnings, but some results mentioned calorie information.  Results: Study 1. The eye tracking indicated that price, products, and brand/logo (e.g., favourite brand) were attended the most by shoppers/respondents when purchasing alcohol. Those messages with medium risk (calorific value information) were holding respondents' gaze for longer than low (unit information) and high-risk messages (health warning)- with minimal difference between high and low. There was a lack of attention and a brief mention of calorie information on alcohol products by the respondents, as some recommended calories on alcohol products, however, they discussed more about health warnings.  Study 2. Respondents were influenced by price, brand, and alcohol percentage. Respondents stated that a positive and helpful element of current labels is text-based information rather than symbols, and a negative aspect is that they don’t stand out. They said the positive for calorie mock label was that it raised awareness providing connection between alcohol consumption and calories. They also said that the negative about calorie mock label was that it was inadequate. The paper concluded that respondents in the mock shopping tasks had little attention to labels and thought they were not specific, relatable, and bold, and more attention was paid to brand and price. |
| Vecchio, Annunziata and Mariani 2018 | Italy | Wine | Quantitative research; Artefactual field experiment; within subjects design. | n=103  21+  Consume wine at least once a week  51% female  average age of respondents 29.12 | Aim: Examined respondents’ preferences and needs for nutrition information on label and off label.  Results: More than 60% of respondents are health orientated. 36% of respondents self-reported low awareness of wine nutritional knowledge. Low awareness for the link between obesity and the consumption of alcohol. Believe wine in moderation has beneficial effects on health. 24% reported they never looked for nutritional information for wine. However, they support and are interested in nutritional labelling on wine. 45% of respondents read nutritional information when buying food or non-alcoholic beverages. Preferred traditional information to be on manufacturing companies’ websites, and public information sites, whereas their least preference was for innovation like label QR codes or smartphone apps (in reference to receiving nutrition information via alternative sources). They would mostly prefer a nutritional panel label which is positioned on the back of the bottle. The lowest preference was given to the label with less information (without nutrition information) with a website recall link. Females read nutrition labels more; they are also willing to pay for those nutrition labels that are more detailed; they seek information when making a choice as well as based on the health evaluations. Higher health interest is connected to preferences for more informative labels. For respondents with higher involvement with wine, there was a decreased preference for nutrition labels. Overall, respondents were interested in nutrition labels (nutritional panel label). |
| Walker et al. 2019a | New Zealand | Beer, wine, ready-to-drink (RTD), spirits, and sparkling wine | Qualitative Research; Seven focus groups exploring different labels. | n=35  18+  Purchased and consumed alcohol at least one alcohol product during the month  Group 1: 18-25 years, mild/moderate drinkers  Group 2: 18-25 years, heavy drinkers  Group 3: 26-50 years, mild/moderate drinkers  Group 4: 26-50 years, heavy drinkers  Group 5: ≥51 years, mild/moderate drinkers  Group 6: ≥51 years, heavy drinkers  Group 7: Māori-only, mixed age, heavy drinkers | Aim: Explored respondents’ awareness and views of energy alcohol labelling; the effects of it on alcohol purchase and consumption. Results: Respondents identified that calories, kilojoules, standard drinks, and % daily intake were not well understood and confusing apart from health-conscious respondents. Respondents were unaware of the energy content in alcohol products and that the main energy source is alcohol itself, believing it was sugar; they underestimated the energy content of red wine and overestimated the energy content when it comes to beer. They understood that a link exists between weight gain and the consumption of alcohol. Most found nutrition information panels overwhelming, difficult to understand, and not useful. Energy content information was viewed as most likely to influence their decisions when purchasing or consuming alcohol products when trying to choose between different products, or they were motivated to limit energy consumption. The preferred and favoured label was combined front label; the energy icon/label (kilojoules, calories, and % daily intake), standard drinks, and % alcohol content; included all 3 in one single label. Although this label was preferred, it would not influence consumption and purchase, hence, they did not see it as ideal. Most stated that labels presented would provide little or no impact on their alcohol consumption and purchasing behaviour. They stated their ideal label would include both front and back (simple on the front and detailed on the back), easy to understand, with a nutrition information panel-even though they did find it less useful previously. In general, there was a preference for enhanced labelling and more information, and not only energy content, as they would also like to see allergy and ingredients. Respondents felt that the energy labels should be on the front of alcoholic beverages. It should be simple, easy to look for, engaging visually, and not look like an award label for wine. In order to have an impact on behaviour, it should also have an easier format to allow comparison between different beverages. |
| Walker et al. 2019b | New Zealand | Beer, wine, spirit | Quantitative research; randomised controlled trial. | n=615  18+  58% female  mean age 41.2 years  78% met criteria for heavy alcohol use  Purchased and consumed alcohol at least one alcohol product during the month | Aim: Examining effects of energy alcohol labels on respondents’ purchase behaviour and attitudes.  Results: The majority of respondents were of the view that food and non-alcohol product labels were important; 86%, useful 78%, and 42% thought the labels were easy to understand. 52% thought that energy labels should be presented on alcohol products. Attitudes were not affected except that respondents thought that the back nutritional information panel (NIP) (label 1) and interpretive (energy content-kilojoules/calories with an orange stopwatch icon showing the amount of exercise needed to burn off the presented energy) (label 3) labels compared to control (label 4) were more expensive. 55% of respondents assigned to the control label (label 4) and 53% who were assigned to the NIP (label 1) believed that the back of the bottle was the most preferred place for the energy label. The front of the bottle preference by itself or combined with the back of the bottle was highest for those assigned to combined (label 2 on the front, three components: energy content-kilojoules/calories, standard drinks and % alcohol content) 42%, and (interpretive label 3) 43%, compared to NIP (label 1) 21%, and control (label 4) 28%; the most preferred place was still the back of the bottle. Although there is support for energy and nutrition label information, there was no reduction in purchasing and consumption of alcohol products by the presented energy labels. The NIP (label 1) and an interpretive (label 3) (for Māori respondents) increased the purchase, creating a negative impact, compared to no label; overall, the purchase was increased by some respondents. Despite not impacting the perceived energy content of alcohol products, there was an increase in respondents' confidence, ability, and accuracy in estimating the energy content of alcoholic beverages. This was much increased when made in calories and not in kilojoules. |
| Winstock et al. 2020 | United Kingdom | Alcohol products | International cross-sectional survey; quantitative. | 75,969n respondents from 29 countries/regions  Used alcohol in the last 12 months  16-85 sample age  mean 27.0  2/3 males | Aim: Explored perceptions of seven health warnings on alcohol-related health harms such as cancer, whether respondents believed these messages, were aware of them, found them personally relevant, and whether they would reduce alcohol consumption. The study mainly focussed on health warnings, but one of the health warnings was related to calorie information “a bottle of wine or 6 bottles of beer contain as many calories as a burger and fries”.  Results: 36.1% of respondents reported that calories information was new to them. Males, and under 25 years said that calorie information was new to them. The calories message was 79% believable, and personally relevant, 28.9%. Females believed the calories messages 81.4% more than males 77.6%. 28.9%. of respondents found calories message personally relevant. Cancer message was found to be the highest for making them consider drinking less/changing behaviour as 39.6% reported; and for the calories 28.4%. Females 39% compared to males 22.5% more likely responded to calories, and also cancer, health myth, and violence labels to consider their drinking reduction. For many of the messages, except calories, heavier drinkers reported that they would consider drinking less. Older respondents, compared to younger ones, believed nearly all the label messages. Over 25s stated that calories message would make them drink less, whereas under 25s preferred liver, heart, violence, cancer, and health myth messages. Overall, personal relevance was found to be really important for all messages; the text violence label (negative frame) was found to be the most believable and personally relevant, and the text cancer label (specific/positively framed) was most likely to make respondents consider drinking less. |
