# Supplementary material for: Consumers’ Attitudes and Preferences Towards Ingredients List, Nutrition Information and Health Warning Labelling on Alcohol Products: A Scoping Review
Source: Curr Nutr Rep. 2026 Jul 22;15(1):62. doi: 10.1007/s13668-026-00784-y (PMC13391450; doi:10.1007/s13668-026-00784-y)
Supplement: Supplementary file 4 — Supplementary Material 4 (DOCX 27.5 KB) [file 13668_2026_784_MOESM4_ESM.docx]

**Appendix 4: Table II Summary of articles included in review that examine the ingredients list**

| **Author/Year** | **Country** | **Type of Alcohol** | **Study design/ method**  **according to the study authors** | **Population sample** | **Outcome/ Comments** |
| --- | --- | --- | --- | --- | --- |
| GFK. Consumer Insights Report 2016 | Germany, Poland, Denmark, Netherlands, Spain, United Kingdom, Italy, France Romania | Beer, wine, spirits | Quantitative Research; Online survey with random sampling approach. | n=9,008  18 - 65 years old  Majority female  Denmark n=1001;  Poland n=1001;  Spain n=1001;  Netherlands n=1002;  Germany n=1001;  United Kingdom n=1001;  France n=1002;  Romania n=1000;  Italy n=1000 | Aim: Examined consumers’ call for all alcoholic beverages to receive the same ingredients and the full nutrition information per 100ml as for other products.  Results: 86% of respondents believe they should have access to the same ingredients for alcoholic beverages (wine, beer, spirits) as they currently receive for any other drink and food product; up from 74% in 2014. 74% of respondents are interested to see ingredients list information, highest in Italy (93%) and lowest in the Netherlands (44%). The more alcohol people consumed, the less interested they were in the information, similar for those who drank more regularly than infrequent drinkers. About one third would focus solely on one information source, whilst 57% would use two or more, a mixture of digital online sources (such as applications and websites) and traditional information sources (such as labels, advertising, and in-store communication) to obtain information, 45% used a mixture of digital online and traditional offline sources, 31% would use traditional, and 16% digital. 75% of respondents would use brand-supported sources to access ingredient information, such as brand labels or websites. When observing the label used as one of the sources of information, 69% would use the label (still the most popular but not the only source of information) as a source to access the information. When observing the label used as one as the only source to access the information, 22% would use the label. |
| Grunert, Hieke and Juhl 2018 | Denmark/Belgium  Six European  countries: Denmark, Germany, Netherlands, Poland, Spain, United  Kingdom | Beer, wine, gin, vodka,  whiskey and rum | Quantitative Research; Online survey using random sampling approach. | n=5,395  18-65 years  Denmark n=787 (51% male); Germany n=993 (51% male); Netherlands n=934 (54% female);  Poland n=1003 (51% male); Spain n=810 (56% male);  United Kingdom n=868 (54% female) | Aim: Examined to which extent ingredient and nutrition information from off-label sources is wanted and used by respondents, how this is affected by health interest, involvement with the product and previous knowledge of this information.  Results: Respondents had a medium level of interest in ingredients information when it comes to information wants but low levels of interest when it came to use. Spain had the highest interest in ingredients information and usage across all sources, and the Netherlands, followed by Denmark, had the lowest. Most interest in obtaining information was from public and health websites, in-store, and least interest from apps and advertising. Older respondents were more interested in ingredients. Males are more interested in the product, whereas females have more health interest, and it becomes stronger with age. The study suggested that respondents' want and use of ingredients information was driven by having a general interest in the product more than for health reasons. |
| Moore 2010 | United States of America | Beer, wine distilled spirits | Quantitative and qualitative research; Survey 1: Opinion poll survey;  Survey 2: National randomised survey;  Survey 3: Follow-up telephone survey. | Survey 1  n=1,042  18+ years  Survey 2  n=503  18+ years  Survey 3  n=1,003  21+ years | Aim: Survey 1: Opinion poll to explore consumer support for change in labelling information.  Results: General public supports mandatory ingredient labelling. There is also support for information about allergies.  Aim: Survey 2: Determined what type of labelling information respondents think is helpful.  Results: 90% of respondents supported mandatory labelling on all wine, spirits, and beer beverages. 79% agreed that these need to include nutrition and ingredient information and did not believe that having a label without complete nutritional and ingredient information was beneficial. The support for ingredient labels did not differentiate between political associations, use of alcohol, or demographics.  Aim: Survey 3: To identify what alcohol label information respondents consider most important.  Results: Main findings related to nutritional information, and it outlined overall that respondents are in favour of label information that is complete as it is needed to allow ingredients about product formulations, calories for weight control, moderate drinking advice, and products comparing information |
| Pabst et al. 2021 | Australia  Germany  Italy | Wine | Quantitative Research; Discrete choice experiment; Random selection; between and within subjects. | n=745 Australia (51% male)  n=716 Germany (57% female)  n=715 Italy (52% female)  Had to drink wine at least once a month | Aim: Examined labelling of nutrition and ingredients on wine choices of respondents and their reactions toward labelling.  Results: Unless respondents were positively or negatively reminded of the ingredients use information they paid little or no attention. Ingredients information only received positive preference from respondents in Italy when choosing wine and their preference was for short ingredient lists, whereas, in Germany and Australia, respondents were not in favour of ingredient labelling. However, respondents in all three countries when making their purchasing wine decisions had low attributes/concept importance towards the ingredients list. Respondents would pay more attention to the ingredients labelling when there is negative media information published about wine ingredients (such as the use of ingredients other than grapes), and this would influence their preference which would be without ingredients list (clean or natural) or only shortlist. Only in Germany did positive and negative information increase ingredient preferences, whereas, in Australia and Italy, it was only negative information; thus, negative media coverage influences respondents' attention more than positive media coverage. In Germany and Italy, respondents reacted strongly as they would not buy any wine in the context of negative media coverage towards ingredient labelling. |
| Pabst, Szolnoki and Mueller Loose 2019 | Germany | Wine | Qualitative Research; Three focus groups and observation of choices. | n=21  11 male  Had to drink wine at least twice a month but not be seen as wine experts | Aim: Examined respondents' reaction to nutrition and ingredient labelling: how important it is, whether there is an influence on wine demand and the effect on respondents' attitudes to wine being a natural product.  Results: When observing the ingredients label information for the first time, there was confusion, insecurity, uncertainty, and incomprehension by respondents. Only 1/3 of respondents who looked at the label presented on the back of the bottle noticed new ingredients information. 2 out of 3 did not understand or know the presented ingredients on one of the labels that included shorter ingredient list. 76% were of the view that wine did not have any ingredients except wine and ingredients caused respondents confusion. 13 out of 21 stated that they see wine to be a very natural product and that they have lots of trust towards the wine industry. After being presented with the label with extensive ingredients, there was doubt among 2/3 of respondents that the product was natural. 1/3 of respondents still thought the product was natural, mostly because of 99% wine content. For transparency and fairness reasons, many respondents supported mandatory ingredient list labelling. In the buying decision, the back label plays a minor part, and other aspects, like price, bottle design, occasion, or recommendations, are more important. There was a preference for a shorter ingredients list for 1/3 of respondents; however, most would not reduce wine consumption because of the ingredients labelling. |
| Vecchio, Annunziata and Mariani 2018 | Italy | Wine | Quantitative research; Artefactual field experiment; within subjects design. | n=103  21+  Consume wine at least once a week  51% female | Aim: Examined respondents’ preferences and needs for nutrition information on label and off label. Mainly focussed on nutritional information labelling, but one result mentioned ingredient list information.  The study briefly mentioned and asked consumers whether they were interested in receiving a mandatory ingredient list on wine labels. They were less interested in the ingredient lists than in nutritional information. |
