# Supplementary material for: Consumers’ Attitudes and Preferences Towards Ingredients List, Nutrition Information and Health Warning Labelling on Alcohol Products: A Scoping Review
Source: Curr Nutr Rep. 2026 Jul 22;15(1):62. doi: 10.1007/s13668-026-00784-y (PMC13391450; doi:10.1007/s13668-026-00784-y)
Supplement: Supplementary file 5 — Supplementary Material 5 (DOCX 77.9 KB) [file 13668_2026_784_MOESM5_ESM.docx]

**Appendix 5: Table III Summary of articles included in review that examine the health warnings**

| **Author/**  **Year** | **Country** | **Type of Alcohol** | **Study design/method**  **according to the study authors** | **Population Sample** | **Outcome/Comments** |
| --- | --- | --- | --- | --- | --- |
| Al-hamdani and Smith  2015 | Canada | Wine, beer and hard liquor | Quantitative Research; mixed design; within and between subjects;  1) Online product-based and consumer-based perception scale: 2) Online questionnaire. | 92n respondents  66.2% female  Mean age 36.4 | Aim: To see if alcohol labelling based on lessons from tobacco labelling have an effect on consumer perceptions.  Results: Regarding positive product and consumer-based perceptions measures, respondents found bottles with warnings less positively than the standard ones. Combined strong text and image warnings on bottles decrease/have less positive product and consumer-based perceptions for respondents. Exposure to combined text and image warnings on a plain wine bottle increased the chances for respondents’ warning recognition. Two unexpected findings based on their hypothesis. One was that respondents only had less positive product-based perceptions of text warnings presented on the hard liquor suggesting this form is less effective. Secondly, combined text and image warning labels had greater recall compared to text warning labels alone on plain bottles. Overall, combined text and image warnings and plain labelling had the greatest effect on respondents. Plain labelling increased health warning recognition. |
| Annunziata et al. 2016a | Italy, France, Spain, United States of America (USA) | Wine | Quantitative Research; Cross-country online survey; conjoint design. | 1016n respondents 330 in Italy;  185 in France;  195 in Spain;  306 in USA (east coast)  51% female  European 18-70 years old, drinking wine at least once a month USA 21-70 years old, drinking wine at least  once a month | Aim: Analysed respondents’ behaviour, label use and belief, interest in health warnings, wine labels information, and their preferences for additional information.  Results: Health warnings were found more useful in European countries. “Ban on alcoholic beverages to children under 18/21 years” and “do not drive after drinking” health messages were preferred in all countries compared to “avoid drinking alcohol during pregnancy,” which had greater importance in France and USA. USA respondents also placed a higher value on “avoid drinking alcohol when you are taking medicines” and “alcohol increases the risk of violence” than the European countries. Respondents preferred information on the front label than the back label. Italian and Spanish respondents’ preference was the full version logo and statement for the health warnings, whereas French and USA respondents’ preference was the simplified version only with a logo. When undertaking the segmentation of the total sample, there were 4 cluster groups. There were differences between these groups in their interest regarding receiving further information on the wine labels. For example, 1st cluster (22% of the sample - mainly females (61%) aged 35-55 years with higher education with greater USA respondents) preferred nutrition information over health warnings. 2nd cluster (35% of the sample - mostly females under 45 with the highest concentration of respondents with children under 16 years) gave higher point to health warnings over nutritional information and read the front label. 3rd cluster (28% of the sample - mostly men over 35 years (62%)) gave most importance to health warnings, then an indication of a number of units not to exceed for consumption. 4th cluster (15% of the sample – more frequent consumers of wine) preferred indication of units not to exceed followed by health warnings. Although there is a difference in clusters, overall, respondents do support health information on wine labels. Implementation costs should be taken into consideration. |
| Annunziata et al. 2016b | Italy | Wine | Quantitative Research; Online survey. | 300n respondents  51% female  18+ years old  Consuming wine at least once a month | Aim: Investigated respondents’ preference and interest toward wine health warning labels and sociodemographic characteristics.  Results: Respondents were interested in health warnings on wine labels, with 62% preferring the logo together with the claim. Many believe that alcohol, particularly wine, can have beneficial effects on health if consumed in moderation. 55% found it useful to receive health and nutrition information on the label. 52% identified having warnings regarding possible side effects of excessive consumption. 40% of respondents read the front label, 16% stated they rarely read it and 11% only if buying for the first time, and 9% never read it. Only 26% read the back-label information. Econometric model findings outline a variety of factors that influence health warnings interest, such as consumption and sociodemographic characteristics. Females, younger respondents, and those health awareness-orientated appear more interested in health warnings labelling. Heavy drinkers are least interested. 38% think “do not drive after drinking” warning and 33% “avoid drinking alcohol when you are taking medicines” warning are most useful. |
| Annunziata et al. 2016c | Italy | Wine | Quantitative Research; Questionnaire Survey; Conjoint design. | 300n respondents  51% female  18-75 years  Italy:  28% Campania,  27% Emilia Romagna,  25% Lombardy,  20% Lazio | Aim: Analysed respondents’ preferences and interest in health warnings and nutrition on wine labelling.  Results: 55% reported it would be useful to receive more health characteristics information of wine through the label, and 8% thought it useless. 40% stated that they read label information, while 6% do not read it when they choose a bottle. When it comes to the back label, 26% read it, and 12% said never. Many believe that alcohol, particularly wine, can have beneficial effects on health if consumed in moderation. A conjoint analysis found that 30% preferred a health warning label with a logo and statement together. There were 3 different consumer segments that cluster analysis identified: 1) detailed information seekers: 25% of respondents, mostly females with high wine involvement, high interest in health warnings (28%), and pay attention to the back label. 2) health warning seekers: 48% of respondents who were mainly younger men (under 44) and had a high interest in health warnings, preferring the version with a logo (30%). 3) 27% of respondents, classed as simplified information seekers (aged 45-54): with a relatively low educational level, includes respondents that attached the least interest in health warnings. |
| Annunziata, Vecchio and Mariani 2017 | Italy | Alcoholic beverages | Quantitative Research; Questionnaire survey. | 385n respondents  54% male  18- 30 years old  Mean age 22.4 | Aim: Analysed university students attitudes and interest towards alcohol health warnings.  Results: 48% of respondents paid irregular attention to the alcohol label information, and 28% stated to never read them. They thought that there was too much information and too many warnings on labels. 32% stated they noticed the “drink responsibly” statement at least once as it has more visibility but had minimal effect on behaviour. 27% noticed the logo “do not drive after drinking”. The statements most preferred were “do not drink while taking medicine” and “do not drink and drive”, while less preference is for foetal risk and brain damage that are considered long-term effects. 82% thought that a negative specific/direct-text/logo message (shown on the back label) that was a logo with a car that was wrecked instead of a generic one had more of an emotional effect. Cluster analysis showed 3 groups with different attention and preferences in relation to the type of warning. Cluster 1; 32% younger respondents with a moderate consumption pattern, who drink for pleasure and fun and are not fully informed on risks, only occasionally pay attention to warnings on labels and are doubtful about their usefulness in influencing their choices. Cluster 2; 40% respondents (26-30 years) mainly drink on special occasions with a ‘responsible attitude’ toward alcohol are more aware of the social and health risks, reveal a positive attitude towards health warning labels and pay greater attention towards them. Cluster 3; 28% respondents (mostly men 18-20 years) who frequently drink heavily and are mainly motivated by imitating friends are more likely to be uninformed about social and health risks associated with alcohol consumption. They do not see health warning labels as useful or relevant, paying no attention to them. |
| Annunziata et al. 2019 | Italy and France | Wine | Quantitative Research; Discrete choice experiment using online survey. | 500n respondents  Generation Y  Defined in paper as born between 1978 and 2000  Females dominated 53% France;  60% Italy  The average age of French respondents 23.3 years  Italians 25.2 years | Aim: Analysed Generation Y preferences, attitudes, and interest towards health warnings wine labels.  Results: 28% of French and 38% of Italian consumers declared that they have never noticed health warning labels. French consumers found warning labels less effective than Italian consumers as there was no effect on their behaviour. 60% of Italians and 50% of French do not remember any warning labels. Respondents preferred not to have logos or messages on the labels. However, if a message was present for both countries, the preference is for neutrally/positive framed messages instead of negative ones. If warnings were used, the preference is for the short-term effect “do not drink and drive” over the long-term effects of alcohol on the brain. The warning label should be small and on the back of the bottle, and neutrally framed. There were differences between countries- Italians were willing to accept a short-term neutral message logo with negative consequences of drink driving and give more importance to the warning message than the position of the logo. French do not prefer any of these messages, short-term drink driving and long-term brain damage, and if there is a message, the position of the logo is more important, that is, the back, and for the message not to be negative. The paper suggested that warnings are more effective if on posters, signs, or advertising compared to wine labels. |
| Annunziata et al. 2020 | Italy and France | Wine and beer | Quantitative Research; Discrete choice experiments using online questionnaire consumer survey. | 659n respondents  Millennials  Defined in paper as born between 1978 and 2000  394n Italy and 265n France  Well balanced between male and female  Mostly  composed of the younger segment of Generation Y | Aim: Analysed alternative health warning formats on respondents’ beer and wine choices.  Results: Choices for beer and wine of young respondents are influenced by the design, framing, and visibility of warnings and are driven and influenced by the type of alcoholic beverages. The acceptance of warnings is higher for beer than for wine. Respondents have a higher preference for a logo on the neck of the bottle with a neutral message for beer (about the risks of drink and driving- short-term effect); and for wine on the front label and without a warning message. Negatively framed messages reduce respondents’ preference for both alcoholic beverages. Warnings regarding the long-term effects of alcohol on the brain decreases respondent preference, and they preferred short-term effects messages. There was no clear preference for the logo size. The study found that health warning effect choices and preferences (framing, visibility, design) are influenced by different beverages. Latent class modelling was undertaken to identify preferences. For beer: Class 1 (28%) mostly males, not frequently drinking, claiming warnings have no impact on behaviour, preference for a bottle of beer without logo; Class 2 (24%) heavy drinkers, prefer “drinking and driving” warning on the front of the label and worry about alcohol consequences; Class 3 (22%) moderate drinking, a higher number of older respondents, positive preference to both logos but prefer “brain damage” one; Class 4 (19%) French frequent drinkers, do not want any warning; Class 5 (7%) mostly females, not involved in risky drinking behaviour/decrease drinking when they see health warning, do not want the negatively framed message. For wine: Class 1 (35%), mostly French males, do not drink frequently, but when they do, they drink too much, logos “decreases their utility”, and “brain damage” more than “drinking and driving” one, not concerned about negative alcohol risks; Class 2 (33%) younger females, frequently consume alcohol, prefer no logo but if there is one the “drink driving” (small one on the front), prefer no negative messages, do not think warnings have effect; Class 3 (19%) older respondents, prefer to see warning logo “drink driving”, neutrally framed message; Class 4 (13%) older respondents, want front label logo, low alcohol content, think about alcohol risks. The two biggest groups (Class 1 and 2) considered warnings ineffective. Older Millennials who have moderate consumption are worried about the side effects of alcohol, and their choices tend to be influenced by warnings. On the other hand, younger Millennials are not. It seems that overall, Millennials are not that concerned about alcohol negative effects. The suggestion is that using QR codes may be used to minimise overstimulation on labels. |
| Blackwell et al. 2018 | United Kingdom | Alcohol | Quantitative Research; Online between-subjects experimental study; Online survey. | 1884n respondents  Equal split male and female  At least 18 years of age  Mean age 35  Report drinking alcohol | Aim: Examined the health warnings and unit labels and assessed the understanding, intentions, motivation, and attitudes of respondents’ towards drinking and examined the best methods to deliver the label information.  Results: After viewing the labels, the respondents found that the accuracy of estimating weekly alcohol serving limits was greater for those that looked at the novel labels designed for the study than the current labels used by the alcohol industry. The unit label type did not have much impact on respondents' consumption reduction, drink choice, and health. Support for labelling policies has shown a main effect of label type greater for the calorie information than the health warning, which received the lowest support. Looking at the content, cancer v mental health; there was higher motivation to drink less and avoidance for those randomised to cancer warning compared to a mental health warning. Looking at the general v specific, those randomised to specific warnings had less reactance, more believability, and higher response efficacy than those assigned to the general warnings. Negative v positive warnings, respondents found that for negative ones, there was higher motivation to drink less, reactance, and avoidance than the positive ones. Clear alcohol units per serving may be informative to promote harmful drinking in combination with health warnings. |
| Cho and Rim 2013 | Korea | Alcohol | Qualitative and Quantitative Research; Surveys. | 500n respondents  67.6% male  32.4% female  Majority 30-39 years old  Had exposure to and experience of drinking alcoholic beverages | Aim: Explored respondents alcohol consumption attitudes or behaviour and the relationship between alcohol warnings.  Results: Many respondents alleged that there was low health warnings exposure, as only 2% saw these messages. It was found that respondents’ alcohol warning messages positively affected their attitudes when it came to moderation or reduction of alcohol drinking. Different messages had different results. For example, the effect of negative text aspect messages was slightly higher than positive text aspect messages. Those alcohol warning messages addressing other-related issues (i.e., effects of drinking alcohol on others) were also slightly higher than self-related issues (i.e., caused alcohol consequences to themselves). |
| Clarke 2017 | United Kingdom | Beer or wine | Between subjects design with random assigning. | 162n respondents  84 female  Mean age 22.21  Aged 18-30 years old  Weekly consumption of alcohol | Aim: Tested the influence and the effects of Drink Wise labelled glasses on the consumption of alcohol products.  Results: Respondents were advised to drink in pairs, and those with a closer relationship consumed more. Males consumed more alcohol than females. Even though 85% of respondents read and noticed the labels on the side of the glasses, it was found that on the alcohol consumption in a semi-naturalistic drinking bar laboratory environment, Drink Wise labelled glasses provided no effects on drinking behaviour. 16.25% believed the glasses had an effect, whereas 80% believed that they had no effect on the alcohol amount they consumed. The majority stated they these warnings could be useful in getting the public to consume less, and 14% specified that this was only in certain individuals. It was stated that warning labels provided on glasses seem not to influence alcohol consumption and have no benefit. Alternative strategies need to be provided to encourage behaviour change among younger drinkers, particularly in naturalistic settings. |
| Clarke et al. 2020 | United Kingdom | Beer or wine | Quantitative Research; Between subjects, factorial design, experimental study- randomised. Note image and text also known as pictorial or graphic in this study. | 6024n respondents  Equal male and female  Mean age 49.5  18+ consumed beer or wine regularly | Aim: Assessed the impact of health warning labels on alcohol product selection and respondents’ cognitive and emotional responses.  Results: Health warnings had more effect on reducing alcohol drinking selection than no label- health warning labels with images had more effect than text only. Compared to no labels, all labels increased avoidance and reactance reactions, the highest for the label with an image. When comparing image and text warning v text only, there was a reduction in the selection of alcoholic drinks for the image and text warning, but these labels increased the odds of selection when compared with image only warning. Only 31.74% of respondents stated labels to some degree acceptable and in terms of acceptability, labels with images were rated the least acceptable (text-only 37.33%, image-and-text 34.18%, and image-only 23.65%). However, the image-only label was most effective in reducing alcohol drinking selection; image-only (49%), image and text (56%), text-only (61%), no label (77%), and there was the largest effect among all the labels. Health warning labels that depicted the risk of cancer had a greater effect on reducing the selection of alcoholic beverages. |
| Coomber et al. 2015 | Australia | Alcohol products  Main alcoholic  drink was categorised into beer, wine, spirits, pre-mix, cider, and other (unspecified alcohol beverages  and home-brew beer) | Qualitative Research; Online survey. | 561n respondents  Majority male  18–45years  Mean age 33.6 | Aim: Examined awareness of the voluntary warning labels and the respondents’ use of DrinkWise website .  Results: The study looked at recall and recognition; no respondents freely recalled the “Get the facts” logo, and a quarter recognised it. Best recall was for “it is safest not to drink while pregnant” message (16.1%) and recognition was 34.3%. Respondents' highest awareness was for pregnancy labels, and respondents with higher education were more aware of the “kids and alcohol don’t mix” message (younger respondents were less aware of this message). Only 7.3% had visited the DrinkWise website, and those who recognised the “Get the Facts” were over 7 times more likely to visit the DrinkWise website than those who did not. Respondents that were more likely to have an awareness of the labels were younger drinkers, those that drank directly from a bottle or a container, those with increased binge drinking frequency, and those who were in support of health-focused warnings. Respondents were more likely to have positive recognition of the “Get the Facts” logo associations and visit the DrinkWise website if they drank directly from a bottle or a container, had increased binge drinking frequency, and those that were in support of health-focused warnings. The website is used more by binge drinkers, those that consume from cans or bottles. Thus, binge drinking frequency increased logo exposure and label as well as website visiting. Older drinkers, compared to those 18-24 year-olds, were less likely to have recognition and awareness of the warning label. Despite younger drinkers having more recognition of labels and logos, they were not more likely to visit the website DrinkWise. Females were less likely to visit the Drink Wise website. Those who drank spirits and wine were more likely to visit the website than beer drinkers. Australian warning labels are not well known, and there is minimal use of the DrinkWise website by respondents. |
| Coomber et al.  2017a | Australia | Alcohol products | Qualitative Research; Four focus groups. | 26n respondents  58% female  18-25 years  Respondents consume alcohol | Aim: Whether pictorial or graphic alcohol warning labels are effective as an intervention for reduced alcohol consumption harms among respondents and their attitudes to them.  Results: Increased size and placement of warning labels made them stand out. Respondents preferred these on the back label, though. Respondents were of the belief that the warnings were not relevant to their current lifestyle, and they disregarded them; they acknowledged that the “breadth” of the warning message would be of value to others. Respondents stated that they would become habituated to them, and once “inebriated”, they would have no effect. They thought that pictorial messages were conveying, and even though graphic warnings had stronger responses, they also thought they were not realistic. Respondents preferred pictorial (drawn) warnings instead of graphic (coloured photographs) ones and also liked targeted statistics messages combined with the drawn photo images. Graphic warning (using photographs) was modelled on those used for tobacco products, and the pictorial (drawn images) of the Foundation for Alcohol Research and Education FARE (graphic warnings were also modelled on FARE). Respondents had negative responses to both alcohol labels but also stated that they would avoid both the picture and graphic warnings labels, and these labels would not stop their alcohol consumption. While the warnings increased respondents understanding and thinking about the alcohol use consequences and may elicit change due to their confronting nature, they were unlikely to change respondents' drinking habits. |
| Coomber et al. 2017b | Australia | Alcohol products | Quantitative Research; Online (web based) survey. | 1,061n respondents  50% male and 50% female  18-45  Mean age 33.2  Respondents consume alcohol | Aim: Australian study investigated the short and long-term awareness of alcohol use consequences among adult drinkers.  Results: There were low awareness levels for a number of both short-term and long-term alcohol consumption consequences. For example, it ranges from the short-term “lack of coordination and slower reflexes” 69.3% to the long-term “breast cancer” 15.6%. It was stated that warnings increased awareness for long-term messages “harm to unborn babies” 67.3% and “cirrhosis of the liver” 61.6%. There was more awareness of the short-term consequences 60% in general, for example, “reduced concentration” 69%, “traffic accidents” 68.5%, and “injuries” 60%. Females were more likely to have awareness for both short and long-term alcohol consumption consequences. Those who had higher education were more aware of the long-term consequences. There was more awareness of injuries caused by alcohol for those from mid and high socioeconomic status. High-risk drinkers were less likely to agree that the consequences of alcohol use are traffic accidents, and they were also less aware of the long-term consequences. |
| Coomber, Hayley and Miller 2018 | Australia | Alcohol products | Qualitative Research; Six focus groups. | 40n respondents  22n females  18–25 years  Alcohol-consuming male and female (55%)  Sample categorised as risky drinkers | Aim: Examined respondents' reactions, thoughts, impressions, and perceptions of current alcohol warnings.  Results: Respondents reported rarely hearing about DrinkWise and had a low understanding of it being industry funded. Respondents' impressions about warnings were critical; their awareness was low. Most stated that they had never noticed health warnings before. Respondents saw health warning labels as being there to avoid litigation rather than reduce harm. Those who were familiar recalled a pregnant woman pictogram that raises a glass of wine to her mouth with a strikethrough. They also believed that warnings were hard to find, too small, vague, lack of impact and meaning, the message was weak, unconvincing, and did not deter them from high-risk drinking. Respondents believed that warnings would not influence and encourage them to change their alcohol consumption behaviour, to look for further alcohol harm information (including the website), and the warnings will not prompt discussion with others. |
| Dossou, Gallopel-Morvan and Diouf 2017 | France | Spirits/vodka and wine/champagne | Qualitative Research; In-depth interviews. | 26 respondents  Equal split male and female  15–29 years | Aim: Explored the effectiveness of two mandatory French health warnings, “alcohol abuse is harmful” (text message on the bottom of alcohol advertisements) and pictogram logo regarding pregnancy information on the back of alcohol bottles.  Results: For the text warning, there was higher awareness, noticeability, and recall than for the pictogram warning. 2/3 recalled the text warning. In general, there was a lack of noticeability and visibility for both of the currently displayed warnings; the pregnancy pictogram and the text one, “alcohol abuse is harmful”. The reason is that the respondents are so familiar with them that they no longer notice them. It was stated that both warnings: lacked credibility, marketing elements caught more attention, and were found to be vague and ineffective in making respondents concerned and influencing consumption behaviours to change. Younger respondents were not able to understand the text advertisement clearly. Respondents that were low to moderate drinkers were more likely to be concerned and respond to the messages than younger respondents and heavier drinkers. These warnings had minimal effect on changing respondents’ alcohol consumption as they were perceived as weak. Respondents also felt overexposed to warning labels and were irritated by them. |
| Escandon-Barbosa and Rialp-Criado 2019 | Colombia | Wine | Observational study using eye tracking methods; Hierarchical model, simulated supermarket. | 114 respondents  32% female  67% male  25-54 years old  Consuming at least one glass of wine per week | Aim: Analysed the contents of labels on respondents' (consumption expert/non-expert and gender) buying intentions. Non-expert: low and less frequent consumption, Expert: higher and more frequent.  Results: Label information needs differs between different type of respondents and genders. Showed that to different types of respondents and both genders, the label information is highly relevant as they read this information differently and it does impact their buying intention. Experts consider detailed information relating to the denomination of origin, health warnings, and nutritional information to make decisions, while non-experts can be confused by the detail. However, a combination of health warnings and denomination of origin allows non-expert consumers to have some signal to make a decision. Even though for both females and males, the intention of purchase increases when looking at these three components- for females, it increases significantly- for males, it is high- males do show a greater effect when it comes to their purchasing intention. Females review and use this information more than males when deciding to buy. |
| Glock and Krolak-Schwerdt 2013 | Luxembourg/Germany | Wine | Between-subject and within-subject; two factorial mixed design. | 40n respondents  24 female  Average 23.97 years  Only occasional or light drinkers | Aim: Compare the explicit and implicit attitudes of respondents’ drinking intentions and the effect of different alcohol warning labels.  Results: The warning labels that contradict positive alcohol outcome expectancies/positively related labels (e.g., “alcohol does not reduce your tension”, “alcohol increases stress”) were more effective compared to warning labels that outlined health damaging/negative consequences of alcohol consumption (e.g., “alcohol damages your brain”). Respondents that were shown the contradicting positive alcohol outcome expectancies labels had less positive implicit attitudes after exposure than those respondents that were presented with health-related labels, as these had more positive implicit attitudes towards alcohol. Thus, respondents in the contradict positive alcohol outcome expectancies group post-exposure compared to the health-related group had more negative implicit attitudes, whereas pre-exposure, it was vice versa. The contradicted positive labels were found to be more effective when it came to challenging outcome expectancies, as respondents found fewer social-related alcohol expectancies and higher depression outcomes than those in the health-related warnings group. Respondents also reported that their alcohol consumption intentions tended to be lower post-exposure to positive alcohol-related outcome labels, whereas, for the health-related group, drinking intentions were higher. |
| Hall et al. 2019 | United States of America | Alcohol | Online survey; randomised between subjects experimental study. | 1360n respondents  52% male  At least 18 years  Mean age 37.4 | Aim: Examined respondents’ reactions to health warnings that used the strength of casual language.  Results: Statements that used ‘causes’ (strong language) were more likely to discourage respondents from choosing the product (76.3%) and were supported for implementation by 39% of respondents. ‘Contributes to’ the warning statement came second in discouraging them 13.9%. As the language became weaker, the support reduced, and 66.1% of respondents found ‘may contribute’ (weak language) was the one with the least discouragement; therefore, having the least effect. Cigarette warnings with equal wording that used strong language were viewed in a more positive way than sugar-sweetened beverages and alcohol health warnings. Respondents that rated sugar-sweetened beverages and alcohol warnings were the ones less likely to support ‘causes’ and select it as the most discouraging use than those who viewed cigarette warnings. Overall, the warning statements with strong causal language, such as ‘causes’ respondents’ found most effective, and they supported it as well as ‘contributes to’ as there was support for stronger causal language warning statements. |
| Hall et al. 2020 | United States of America | Alcohol | Online randomised between-within subjects experimental study. | 1352n respondents  52% male  At least 18 years  Mean age 37 | Aim: Examined respondents' behaviour reactions to the text and graphic warnings for alcohol, sugar-sweetened beverages, and cigarettes.  Results: Graphic warnings were found to be less believable, had greater reactance (resistance), and fear, there was lower product appeal, and more thinking about harm. Policy support was also lower for graphic warnings than text warnings, only in unadjusted analyses. Both text and graphic cigarette warnings were more effective and acceptable than alcohol warnings and sugar-sweetened beverages. Reactance was lower for cigarette warnings as there was more fear, message effectiveness, believability, policy support, thinking about harms, and more reduction on the appeal of a product. The study stated that the efficacy statements ‘quit and cut back’ did not change respondents’ self-efficacy to limit consumption and smoking. Respondents who did not smoke, and especially those who consumed more alcohol in the last month, found cigarette warnings more effective. There was no impact on differences in low-income status. Across all three product types, graphic warnings were found to be more effective than text warnings; the effect was small, though. |
| Hobin et al. 2020a | Canada | All alcohol containers (bottles or cans of beer, wine, hard liquor, coolers, or ciders), except local and single-serve beer and cider | Quantitative research; Quasi-experimental design, pre-post surveys. | 1647n cohort respondents  Legal drinking age 19+  Consumed at least one alcohol drink in the past month  Two surveys, 836n at baseline and 811n at follow-up | Aim: Tested the effectiveness of alcohol labels for supporting more informed and safer alcohol consumption among those who drink.  Results: Label warning messages influenced females to drink less; they were more likely to read labels and think about them. Those with higher education also reported reducing their drinking because of warning label messages. Respondents with high socio-economic status recalled label messages more, but they would act less on and change their drinking behaviour. Respondents that were older were less likely to talk to others about warning labels and to think about them. The effects of the enhanced label messages were more found for thinking about them, reading, talking with others about them, and self-reported influence on reduced alcohol consumption. |
| Hobin et al. 2020b | Canada | Bottles or cans of beer, wine, hard liquor, coolers or  Ciders; All alcohol containers, except  select local and single-serve beer and cider | Quantitative research; Quasi-experimental study. | 2049n  cohort respondents  Male majority  Legal drinking age 19+ | This is a follow-on study from Hobin et al. 2020a  Aim: Assessed the effects of alcohol labels in real-world study on consumer attention, thinking, reading, talking to others, and self-reported alcohol reduction.  Results: There was an increase in respondents noticing label changes, thinking, reading, talking to others about messages, and self-reported reduced alcohol drinking. The sample consisted mostly of those respondents who self-reported low levels of alcohol consumption. The strengthened enhanced (alcohol labels such as more prominent, new, serious, and included with the recommendations) messages had an effect, and there was an increase in respondents’ attention, labels information processing, and repeated exposure reduced self-reported alcohol drinking. |
| Hobin et al. 2020c | Canada | All alcohol containers  wine, spirits, coolers, and beer (except select local and single-serve beer and cider) | Quantitative research; Quasi-experiment. | 2,049 cohort respondents  1,056n male majority  Legal drinking age 19+  Consumed at least one alcohol drink in the past month | This is a follow-on study from Hobin et al. 2020a and Hobin et al. 2020b  Aim: Identified the effect of cancer warning labels on recall and knowledge in a real-world setting.  Results: There were three survey waves; only wave 1 included cancer messages, awareness campaign information, and social marketing. The standard drink labels and drinking guidelines were used throughout three survey waves. Respondents noticed labels in all three waves. 2 months following the cancer warning labels being stopped, nearly 25% of respondents exposed to the intervention were able to recall the cancer labels unprompted; when prompted, the recall was 43%. Recall decreased six months after cancer labels were removed. 2 months since the warning cancer label, there was a 10% greater knowledge of alcohol as a carcinogen (breast and colon cancer used in this study) increase in intervention comparative to the comparison site, the increase results were also similar six months after as well. Respondents that were able to recall cancer label information were 2.3 times more likely to know that ‘alcohol can cause cancer’ after also being exposed to media information. Overall, respondents supported that there was a link between cancer and alcohol consumption, and there was an increase in respondents' knowledge and noticeability of the labels. |
| Jones and Gregory 2010 | Australia | Alcohol products | Qualitative Research; Six focus groups. | 44n respondents  23 males; 21 females  18-22 years | Aim: To understand university students' opinions and attitudes towards alcohol warning labels and if these would influence their behaviours.  Results: Most respondents did not believe current warning labels are effective and would not influence their consumption behaviour and attitudes. Respondents are aware of the negative alcohol effects, such as long-term consequences; however, they do not believe them and believe that warnings are irrelevant as they are not likely to affect them until later in life. There is a potential that some respondents would use health warnings to increase alcohol consumption levels to prove their toughness. There was agreement in 5 out of 6 focus groups that the current alcohol warning labels would not change attitudes and beliefs in risk behaviours amongst young adults. Some would rethink their behaviour; however, most declared there would be no effect on their drinking patterns. To influence behaviours, modifications are required to make them more noticeable, specific, format-colour and size, more personal, and concentrate on short-term effects, not long terms such as cancer (which may be ineffective). Alcohol warning label usage should be combined with other strategies and educational measures aiming to change beliefs and behaviours to be of benefit. |
| Jongenelis et al. 2018a | Australia | Beer, wine, spirits/  liquor | Quantitative research; Online survey; Between subjects randomized experiment. | 2087n respondents  Equal number of male/female  18- 65 years eligible  Consuming alcohol at least twice per month | Aim: Assessed whether there were improvements in respondents’ intentions and attitudes when exposed to alcohol cancer information via single v multiple sources.  Results: For both the single and multiple sources, “alcohol increases your risk of bowel cancer” was the most effective message. Alcohol warning messages presented via multiple sources were found more effective than those presented by a single source, as respondents found those messages presented via multiple sources to be more personally relevant, believable, and convincing. There was also greater change among respondents in behavioural intentions from preexposure to postexposure for those presented via multiple sources. Those who received warnings from multiple sources stated that they would and should reduce their alcohol drinking and had reduced intention to drink in a single sitting five or more standard drinks. It was found that the message delivered via multiple online sources (health-related messages presented) provided more changes in respondents’ attitudes and intentions than the same message delivered via a single source (alcohol warning label). A comprehensive approach and using more than just the warning label are required to have an impact; multiple sources should be used. |
| Jongenelis et al. 2018b | Australia | Alcohol products | Quantitative research; Online survey; Between and within subjects randomized experiment. | 364 respondents  72% male  Aged 18–65 years eligible  Average of more than two standard drinks per day | Aim: Investigated whether specific warning messages influenced drinking intentions towards at-risk drinkers and increased their belief in alcohol being a risk factor.  Results: There were increases in alcohol risk beliefs including for diabetes, mental illness, heart disease, and cancer (the liver message did not have significant change). Change in the respondent's alcohol risk belief was largest when they were exposed to messages highlighting the specific disease in combination with alcohol-related harm. The greatest change in alcohol intentions effect was for the statement “warning: alcohol increases your risk of diabetes”. Changes in reduced drinking intentions were also present for messages of cancer and mental illness, except for the liver and heart disease messages, where there were no significant intention changes. The study outlined that warnings outlining alcohol drinking and specific chronic disease may encourage changes in alcohol consumption intentions amongst drinkers associated with long-term harm risk. Warnings alone would not provide behavioural change- potentially considered with other programs and campaigns. |
| Kersbergen 2017  Thesis | United Kingdom | Beer, cider  Alcohol products | Thesis – multiple studies, only those relevant reported; Qualitative and Quantitative Research;  Study 3 comprise three components  Study 3.1 Between-subjects online study:  Study 3.2 Between-subjects online study:  Study 3.3. Within-subjects laboratory study;  Study 6 Focus group. | Study 3.1.  284n respondents  18+  Consume alcohol at least once a month  203 female (71.5%);  Study 3.2.  109n respondents  +18, drink more than 14 UK units per week;  Study 3.3  30n respondents  18+  (53.3% female)  Drink more than 14 UK units per week.  Study 6  13n respondents  (77% female)  18+  Drank at least 10 UK units/week | Study 3; Aim: To compare current alcohol warning labels with novel labels (health-related) on alcohol packaging on drinking intentions effects and how these affected respondents’ willingness to pay for alcohol.  Results: None of the labels had influenced or affected respondents drinking intentions, health concerns, and attitudes. There was no significant difference in respondents' willingness to pay for alcoholic beverages containing new health warning labels. The majority stated that they had seen the current warning guideline label but did not pay much attention to any message labels presented. The novel warnings did not capture more attention than the current ones. When using eye-tracking, the novel/new labels did not attract more attention than the old/current warning, and there was no influence on respondents’ willingness to pay for alcohol, even though the viewing time increased by almost twice when there was an increase in label size, the respondents still found the current labels were more credible than the novel/new ones.  Study 6; Aim: Used focus groups to understand participants impressions of current warning labels and advertising which focussed on responsible drinking then investigated alternatives that would be more persuasive.  Results: Respondents were aware of current warning labels, drinking responsible messages on alcohol advertisements, and alcohol packaging. They did not pay attention to warnings because they were not credible and very prominent. The labels would not influence their drinking behaviour or others, irrespective of the communication means used. They did not find current warning labels, alcohol advertisements, and drinking responsible campaigns to be relevant personally. The message source was also mistrusted by them. Respondents thought that more persuasive messages might be personal if focussed on long-term health outcomes, general short-term alcohol-related harm, and especially messages that reinforce how excessive drinking can affect others. They also thought shock messages might be more effective and powerful. They did not think current messages influenced their or others consumption behaviour. |
| Kersbergen and Field 2017 | United Kingdom | Alcohol beverages containers  Study 2.1  11 bottles/cans of beer, 6 cans of pre-mixed cocktails, 3 bottles/cans of cider, 3 bottles of alcopops and 2 bottles of wine  Study 2.2  cider/beer, large glasses of wine, and shots of hard liquor | Observational research; Cross sectional design; experimental- mixed design:  Study 1 eye-tracking;  Study 2 eye-tracking as in study 1, experimentally manipulated attention. | 60n respondents  Study 2.1  63% female  18+  did not wear glasses  mean age 21.27  Drank an average of 32.12 UK units in the 14 days prior to the experiment  120 respondents- Study 2.2  65% female  +18  did not wear glasses and consumed  (14 units/week for females, 21 units/week for males) | Aim: Investigated respondents’ attention to current warnings and branding on alcoholic products, effects of increased attention; the respondents’ attending to these warnings, motivation, and consumption behaviour.  Results: Study 1. Respondents viewing attention time to alcohol warnings was only 7%, and the attention was when they were less complex and larger in size. More interested in branding than warning labels. Respondents high in motivation to reduce alcohol consumption paid less visual attention to health warnings and branding (alcohol), and their attention was more on the rest of the packaging. Current warnings did not affect respondents' alcohol consumption intentions. Study 2. Respondents with brief intervention paid less attention to branding (this was not specific to alcohol packaging) but did not pay more attention or attended more to warnings. The study manipulated attention to alcohol packaging (the border around the warning label) so that respondents attended to either warning information or the brand. Also, motivation for drinking reduction was not influenced, and it had no influence on warnings attention. For example, those who received alcohol advice spent less time viewing branding information than those in the control group; their reduced attention was not increased to warnings; instead, their attention was increased to the packaging (rest of it). Manipulation to increase warning attention did not reduce respondents’ alcohol consumption intentions. Visual attention to warnings had no effect on respondents’ drinking intentions and did not reduce how much they planned to drink alcohol for the following week. Respondents assigned minimal attention towards alcohol warnings even when they were forced (attention directed) and had to attend to these warnings, with no influence on drinking intentions. |
| Krischler and Glock 2015 | Luxembourg/Germany | Beer and alcopops | Mixed design- between subjects and within subjects. | 122n respondents  84 female  Light drinkers  23.5 mean age | Aim: Assessed the effect of alcohol warnings presented as statements or questions on respondents' alcohol-related outcome expectancies and consumption intentions.  Results: 36.58% of respondents stated that they believe in the effect of warnings. 76.42% said they would be buying the experimental bottles they looked at. All thought that negative/depression-related expectancies were related more to alcohol consumption than those positive/socially related or tension-reduction alcohol expectancies (when answering how alcohol makes them feel). Respondents were contemplating whether picture alcohol warning should be effective, articulated as a statement or a question, and agreed that the question labels were showing some effectiveness as they were able to increase the individual negative alcohol-related outcome expectancies. Statements showed no change and influence in the general outcome and individual positive expectancies, and drinking intentions. |
| May, Eliott and Crab 2016 | Australia | Alcoholic beverages | Qualitative Research; Seven focus groups. | 38n respondents  Aged 18-65 years | Aim: Examined self-identified light to moderate alcohol respondents’ response to the information that ‘alcohol causes cancer’.  Results: Two themes emerged demonstrating respondents’ alcohol-related cancer message resistance 1) that cancer is uncontrollable/unavoidable hence change in behaviour is pointless. The dominant response was ‘everything’ and ‘anything’ and ‘causes cancer’, establishing the resistance to not only the message itself but eventually to change in behaviour. 2) the normalising/justifying alcohol consumption within society as respondents found it a necessary part of their life and they thought they are responsible alcohol consumers. Those 55-65 years old were the only ones talking about drinking in moderation. Respondents found that not drinking was more problematic than drinking due to social obligations and expectations. Respondents associated social obligations and practical reasons (anxiety reduction, confidence increase, networking) with drinking, as alcohol consumption is a necessary portion of life (friendship maintenance and networking). Several spoke about conflicting health information as they were sceptical about the reliability. The paper concluded that respondents demonstrated resistance to the message due to “everything causes cancer”, the cancer being an inescapable disease, and their own social and unproblematic alcohol consumption. |
| Maynard et al. 2018a | United Kingdom | Beer/cider, wine, spirits, alcopops | Quantitative design; Experimental study; Between subject design:  Study 1 online public survey;  Study 2 online between-subjects experiment. | Study 1  450 respondents whole sample  18+  female 54%  male 46%  median age 34  Consumers of alcohol  48 harmful drinkers (65% males- 32 years mean age)  Study 2  1884n respondents  18+  50% male  50% female  median age 35  Respondents consume/drink alcohol | Aim: Investigated attitudes, knowledge, intentions, beliefs, and behaviours impacted by units, calories, and health warning alcohol labels, as well as the best information and presentation of these labels.  Results: Study 1. The majority, 77%, agreed that health warnings were a good idea and there was support for the increased information on alcoholic beverages. 83% of the harmful drinking group respondents supported health warnings; however, 46% would avoid them. 36% of students (18% of the whole sample) were likely to avoid health warnings. Knowledge: When provided with the text health warning, the respondents stated that fertility messages provided new information (37%), followed by mental health (36%), cancer (36%), harm to unborn child messages (20%), liver disease (18%), and driving accidents (17%). Beliefs and attitudes: Respondents believed text messages were true/there was awareness of: the liver disease (95%), driving accidents (95%), harm to an unborn child (91%), mental health (77%), fertility (76%), and cancer messages (72%). After reviewing the warnings provided, 86% accepted the messages compared to 77% at the beginning of the study. However, 28% still stated they would avoid warnings; 20% thought they were annoying, 38% said warnings would control their drinking, and 14% were against the government implementing warnings. Behaviours and intentions: The following messages would make respondents drink less: liver disease risk (39%), drink driving accidents (38%), harm to an unborn baby during pregnancy (37%), cancer (36%), fertility (26%), and mental health (21%). There was support for labels; however, it was lowest for health warnings compared to calories and units.  Study 2. Avoidance and motivation for reduced alcohol consumption were higher for cancer warning messages than mental health ones. Respondents reported lower levels of reactance for specific warning messages, which were more believable, and there was higher ‘response-efficacy’ than a general warning message. Negative message-framed warnings scored higher on reactance, motivation to drink less, and avoidance than the positively framed warnings. |
| Miller et al. 2016 | Australia | Wine, spirits and beer | Quantitative Research; National online survey. | 1547n respondents  72% female  18+ years  The median age 43 years  Regardless of alcohol consumption | Aim: Investigated respondents' responses towards the impact of long-term cancer warning statements on alcoholic beverages and alcohol-related cancer risks.  Results: More than 70% of respondents agreed that all labels presented could raise their awareness of alcohol drinking and cancer risk, and all labels would prompt conversations about cancer and alcohol drinking. Specific cancer statements (3 out of 4 labels had specific messages) instead of general ones were preferred. 50% or fewer agreed that their consumption behaviour could be influenced by the warning message labels presented. 58% stated that the messages would prompt discussion with friends and family. 36% of respondents agreed that the labels would make them drink less often, and 30% would also prompt their friends to drink less. Females agreed with all the impact statements: prompt conversations, raise awareness, educate others about cancer and alcohol risk, and influence drinking behaviour. Those over 43 years were more likely than the younger ones to agree that labels would influence their friends drinking behaviour and would prompt discussions. Those over 43 years, low-risk drinkers (who agreed with the impact statements), and females reported as most likely to read health warnings would be reassessing their behaviour according to the warning, preferred to know the risks and act upon the advice. Males younger than 43 and high-risk drinkers (men were mostly high drinkers in this study) were least likely to agree with the provided statements. The predictor for respondents' agreement and preferences with the impact messages was a high level of being inclined, responsive, and generally attitudes toward warning statements and recommendations. High-risk drinkers found labels less effective in altering their drinking behaviour, and are less likely to read, follow recommendations, and change their drinking behaviour. The study concluded that even though there may be awareness and the labels prompted potential discussion, for the behaviour change to occur, labels should be combined with other avenues and campaigns. |
| Parackal, Parackal and Harraway 2010 | New Zealand | Alcohol containers | Observational Cross sectional survey. | 1129n respondents  Females only  16–40 years | Aim: Reported New Zealand non-pregnant women's preference ratings towards warning labels as a source of information regarding alcohol-related risks during pregnancy.  Results: 65% response rate. 53% of non-pregnant females provided a high rating preference for a warning as a source of information regarding alcohol-related risks during pregnancy. 30% gave a low preference rating. Younger females (below 30, those 16-19) favoured pregnancy warning labels, while those 35-40 were less likely to provide a high preference rating. Compared with abstainers, females that were light drinkers (per day-two standard drinks or less) and moderate/heavy drinkers (per day more than two standard drinks/ less than once per month binge drinking) were less likely to give warnings on alcohol drinking during pregnancy as a source, a medium preference rating—no difference between low and high rating preferences among these three groups. In the study, there were more moderate/heavy drinkers. The study concluded that other prevention methods might also be necessary for behaviour change. |
| Pham et al. 2018 | Australia | Wine | Multi-method experimental design; between subjects; 1^st^ study online survey, 2^nd^ study eye-tracking system. | 1^st^ study  559n respondents  Mean age 31.9  2^nd^ study  87n respondents  Mean age 26.6 | Aim: Investigated the respondents’ attention toward current warning labels and the effectiveness of redesigned labels.  Results: There was a self-report increase in respondents' attention to the optimised design. When warning label presentation was optimised, eye tracking methods identified 81% of the respondents paid attention to labels where changes have been made (colour red and 50% size increase), whereas 59% of the respondents only paid attention to the current warnings/control (black and white). The colour red and a size 50% larger were found to have more effectiveness in gaining attention than the current warnings/control. Respondents viewed warnings for a 0.6-1 second period of 20 seconds total that was shown to them. Overall, there was low awareness as more than 1/3 of respondents didn’t pay attention at all towards the current warning labels, whereas self-reported attention and as per eye tracking, respondents’ attention increased by 37% for the design that was larger and red for the pregnancy warning and DrinkWise in comparing to current black and white/grey label, but there was no significant difference in fixation. However, other sources were included in the study, such as posters, and the study examined only the DrinkWise logo label, not the text statements. |
| Pechey et al. 2020 | United Kingdom | Alcoholic drinks  Beer or wine | Online survey; Between subjects experimental study; mixed -methods Quantitative and qualitative Research; Two online studies using between-subjects designs. | 5528n respondents  Study 1  Self-reported to consume beer or wine at least once a week  50.9% female and  had a mean age of 47.5  4618n Respondents  Study 2  50.7% female and had a mean age of 47.5  18+ years | Aim: Describing the effects and acceptability of image-text (graphic) warnings on alcohol products and energy-dense snack foods.  Results: Presented image-text/graphic warning labels showing bowel cancer produced the highest emotional arousal negative levels and were lowest for the desire to drink the labelled alcohol product. Overall, 25.7% of respondents found health warnings on alcohol acceptable and effective compared to 60.7% that did not (25.5% thought there was effectiveness with warnings to reduce alcohol drinking, and 26.5% found acceptability for warnings). Acceptability was low for graphic health warning labels that were presented on alcohol- 3 of 21 graphic warnings were found acceptable, 13 of 18 energy-dense snack health warnings were found acceptable. Respondents' comments regarding graphic warnings were mostly negative. The most acceptable warnings were type 2 diabetes and liver cirrhosis. The study stated that bowel cancer warnings show the possible potential to reduce consumption; least acceptability. |
| Pettigrew et al. 2014 | Australia | Alcohol products beer, wine, spirits | Multi-method approach; Mixed methods; Qualitative Research; Six Focus groups; Quantitative Research; Online survey. | Focus groups  48n respondents  18-64 years consumed at least 2–3 standard drinks of alcohol per month  Online survey  2,168n respondents  18+ consumed alcohol at least two to three times per month | Aim: Developed and tested alcohol cancer warning statements and tested respondents’ acceptability towards these labels.  Results: Respondents in focus groups expressed their focus is on concise, brief statements and short-term effects preferences; however, most were also aware of liver, cancer, and heart problems. Many believe that ‘everything gives you cancer,’ and there is no alcohol-cancer risk with moderation. Some preferred ‘increases risk’ while others preferred ‘causes’ wording. Most found specific forms of cancer messages more effective instead of referring to general and to have rotating messages, whereas others preferred general messages. Some believed a ‘warning’ word at the front of the messages would help attract attention, while others favoured brief messages and unnecessary words to be omitted. For survey results: those with higher tertiary qualifications, females, and younger respondents found the warnings more believable. Heavy drinkers, compared to lower-risk alcohol drinkers, found messages no less convincing or believable but found messages more personally relevant. Females, younger respondents, beer and wine drinkers, younger high-risk drinkers, and those with higher tertiary education found messages more convincing and personally relevant than males, spirits drinkers, and older high-risk drinkers. General warning received the highest score, and generalised warning message references were found more believable, convincing, and personally relevant than specific message references. The positively framed warning messages were more convincing, believable, and personally relevant than the negative ones. Males find numerical evidence messages more relevant personally than fear ones, and females vice versa. Messages that used the wording ‘increases risk’ were more convincing than ‘can cause.’ The study concluded that positively framed messages and those using ‘increases’ performed better than negative messages and using the wording ‘can cause.’ Positively framed messages were perceived higher; however, the message referred to in a negative form, ‘alcohol increases your risk of bowel cancer,’ performed best of all statements, indicating that specific forms of cancer messages performed better than general ones. Respondents had neutral to favourable responses towards the inclusion of alcohol-label cancer messages. |
| Pettigrew et al. 2016 | Australia | Alcohol products | Quantitative Research; National online survey; between subjects. | 1,680n respondents  50.1% male  18-65 years eligibility  Consumed alcohol at least two days  per month | Aim: Whether designed alcohol warnings to increase the cancer-alcohol link showed utility for effectiveness.  Results: All six presented statements provided favourable outcomes for message attitudes and drinking intention changes. Respondents had more intentions to reduce alcohol consumption after being exposed to the labels (compared to before their exposure), and the most effective message was “alcohol increases your risk of bowel cancer”. The believability and personal relevance was a big factor influencing respondents’ pre to post-change. The high-risk drinkers also had positive changes in their consumption intentions due to all statements, and the greatest effect was for the message “alcohol increases your risk of bowel cancer” followed by “warning: alcohol increases your risk of cancer”. Compared to other respondents’ post-exposure, high-risk drinkers had greater reduced drinking intentions to consume five or more alcoholic drinks in a single sitting. The study concluded that multiple modes of message delivery produce more effective attitudes and consumption intentions outcomes as warnings alone cannot provide substantial changes. |
| Roderique-Davies et al. 2020 | United Kingdom | Wine, cider, beer, spirits and sparkling wines | Mixed methods; Observational study; qualitative; Qualitative Research; Focus groups; 1st study eye-tracker device;  2nd study 3 focus groups. | Study 1  25n respondents  (14 female-11 male)  23-63  18+  Consumers of alcohol  Study 2  10n respondents  (8 females-2 males) mean age 33.9;  members 18+  Consumers of alcohol drinking | Aim: Investigated what label aspects respondents attend to while purchasing alcohol products, including health messages.  Results: Study 1. The eye tracking indicated that price, products, and brand/logo (e.g., favourite brand) were attended the most by shoppers/respondents when purchasing alcohol, and there was little/lack of attention and mention towards health information messages. Those messages with medium risk (calorific value information) were holding respondents' gaze for longer than low (unit information) and high-risk messages (health warning)- with minimal difference between high and low. Some respondents believe they already know the warnings, and these have limited impact. There were suggestions for long-term effects messages such as “mental health” and addiction” and some for short-term such as “concentration, decision making”. Due to the lack of attention to health information presented, it was stated that health messages have very little to no impact on respondents’ purchase decisions. Respondents were interested more in brand and price. 84% had recalled seeing alcohol volume on alcohol labels, 68% unit information, and 40% health information. 64% stated that alcoholic beverages should have health warnings. Study 2. Respondents were influenced by price, brand, and alcohol percentage, whereas they believed health warnings did not impact consumer behaviour. Respondents stated that a positive and helpful element of current labels is text-based information rather than symbols, and even though they are informative a negative aspect is that they don’t stand out. They questioned the efficacy of mock labels and suggested specific ones. Health warnings were not attended to as there was no reference to it, except alcohol by volume. The paper concluded that respondents in the mock shopping tasks had little attention to labels and thought they were not specific, relatable, and bold. Label messages had limited effect on alcohol purchasing or harm minimisation, and more attention was paid to brand and price. |
| Rout and Hannan 2016 | New Zealand | Alcohol products | Qualitative Research; Online survey of pregnancy labels. | 1,488n respondents  387 females aged 18-34  388 females with children under 15 years | Aim: Are current pregnancy alcohol labels effective: assessing recall and awareness by respondents as well as their understanding of the current pregnancy labels.  Results: When prompted visually, more than 4 in 10 can recall at least 1 of the 3 pregnancy labels. When respondents are unprompted, recall of pregnancy labels is low (3%), and after being asked about the labels consideration they have seen, the recall is 10%. Highest awareness of ‘DrinkWise’ text messages (“it is safest not to drink while pregnant”) compared to pictogram message (pregnant lady). Respondents with higher income, better educated, younger, and with moderate or higher alcohol dependence had higher recall for labels. Those with high alcohol dependence also had a higher recall for the ‘Drinkwise’ text message, whereas those with low to moderate alcohol consumption preferred the pictogram. Some respondents, when unpromoted, believed that two text messages meant ‘you could drink alcohol when pregnant’. 67% of respondents stated that a pictogram best shows the link between drinking while pregnant and alcohol harm, whereas 49% stated ‘DrinkWise’ text. Overall, 87% preferred pictograms and found it most effective to tell the message, to prompt respondents' attitudes during pregnancy not to drink alcohol, and for them to have a conversation with others about the risks. 76% thought the message would be even clearer if the pictogram were combined with ‘Drinkwise’ text or the other one. When it comes to red colour, 97% link warning to it. Younger females in New Zealand are most aware of the ‘cheers.org.nz’ website. |
| Sillero-Rejon et al. 2018 | United Kingdom | Beer | Between subjects and within subjects eye tracking experiment; Experimental human laboratory study; online questionnaire. | 128n respondents  50% males  50% females  18+  22 mean age  Regular alcohol consumers who have consumed over the  UK weekly guidelines | Aim: To examine whether enhanced self-affirmation before consumers view pictorial alcohol warnings would decrease defensive reactions, promote positives, and lead to a change in behaviour, and also how the severity of these warnings can influence the reactions.  Results: 47% of the time was spent by the respondents looking at the pictorial labels; however, it was reported that self-affirmation had no effect on motivation, avoidance, reactance, effectiveness, susceptibility, no influence for self-efficacy for drinking less, no difference re visual attention when it comes to highly-severe and moderately-severe pictorial health warnings. Highly-severe pictorial labels produced more reactance and avoidance than moderately-severe and were more effective, and there was more motivation for reduced drinking. There was no impact on perceived susceptibility to the risk revealed between highly-severe and moderately-severe pictorial warnings and no interaction evidence between the severity of the warning and self-affirmation. Some evidence was reported that self-affirmation might decrease warnings, negative reactions, and avoidance among respondents with high levels of self-efficacy to reduce drinking. The study questioned self-affirmation manipulation validity. |
| Stafford and Salmon 2017 | United Kingdom | Vodka | Quantitative Research; Between subjects experimental study- random allocation, control. | 45n respondents  females only  18-25 years  Consumers of alcohol between  2 and 40 units | Aim: Examined the influence of alcohol health warning type on alcohol consumption speed.  Results: Consumption of alcohol was found to be significantly faster with no health warning label category compared to text and pictorial health warnings- these two did not differ significantly. Even though there was a difference in speed of consumption, the alcohol product acceptability had no difference between the text only and the warning label (also no difference between text and pictorial). The pictorial health warnings led to lower ratings of acceptability compared to no health warning. There was an absence between drinking rate influence and protective drinking behaviour for both health warnings. Overall, when looking at whether the speed of alcohol consumption is influenced by the type of alcohol health warning, it was suggested that a warning is more important in influencing the rate of consumption than the type of message. |
| Vallance et al. 2018 | Canada | Beer, spirits, wine | Qualitative Research; Five focus groups. | 45n respondents  36n focus groups  19–65 years  63.9% females  Consumed at least 1  alcoholic drink in the past 30 days  5^th^ focus group 9n | Aim: Respondents’ opinions were explored on acceptability, content, and design of enhanced alcohol labels to provide the best drinking information.  Results: The majority of respondents supported the enhanced labels on alcohol products as they believed that consumers have a right to know and to be better informed about the alcohol consumption risks associated with it and for the labels to be more accessible. They found label information to be important and possibly impactful to the consumer. Some stakeholders were hesitant about the label's effectiveness when it comes to behaviour change, whilst others thought the labels would give consumers a choice and contribute to the intervention of alcohol control. Some respondents thought that labels would be less effective towards certain groups of drinkers, but there was support for the labels to be accessible to a variety of consumers and beneficial in prompting conversations. Respondents and stakeholders were all in favour of both low-risk drinking guidelines and standard drinks, and they felt that displaying these combined in chart and pictogram would be the most effective. The strongest support was for large labels, as it would draw more attention. Stakeholders pointed out that the label location and size would depend on logistical factors such as packaging, bottle shape, and other information placements, as manufacturers would resist large labels that would potentially cover branding. All reported that powerful components of the labels are direct messages that display the link between alcohol-related harms like cancer health messages and those providing awareness for different alcohol-related harms, as well as additional educational campaigns and web links on the labels, to increase information, interpretation, and awareness. |
| Wigg and Stafford 2016 | United Kingdom | Beer, wine | Quantitative Research; Between and within subjects experimental study-random allocation, control; Questionnaire. | 60n respondents  43 females  18-35  Consumers of alcohol | Aim: By measuring respondents’ fear arousal, ‘perceptions of the health risk,’ and their intentions to reduce and quit consumption of alcohol, the effectiveness of the health warning labels was assessed.  Results: There was an increase in intensions to quit alcohol consumption and to reduce the consumption of alcohol for pictorial labels compared to no label/control. However, there were similar intentions for text and pictorial labels, and text and no label/control. Pictorial label risk was higher to no label/ control for ‘perceptions of the health risks of consuming alcohol’ and there was no significant difference in comparing pictorial labels and text ones. The pictorial label was highest for fear arousal than the text label and no label/control. Pictorial labels and text labels were found more effective in increasing intentions to quit and reduce consumption of alcohol and ‘perceptions of the health risks’ than no label/control. There were no noteworthy differences in the main measures of effectiveness between the two different exposures. In general, the pictorial label was most effective as there was higher fear arousal, an increase in perceptions of the health risks associated with the consumption of alcohol, increase in intentions to quit and reduce consumption of alcohol compared to no label/control. Overall, pictorial labels were only slightly higher on intensions to reduce consumption (as there were inconclusive differences in the main effectiveness measures between pictorial labels and text labels). |
| Winstock et al. 2020 | United Kingdom | Alcohol products | International cross-sectional survey; quantitative. | 75,969n respondents from 29 countries/regions  Used alcohol in the last 12 months  16-85 sample age  mean 27.0  2/3 males | Aim: Explored perceptions of seven health warnings on alcohol-related health harms such as cancer, whether respondents believed these messages, were aware of them, found them personally relevant, and whether they would reduce alcohol consumption.  Results: 61.8% of respondents reported that the cancer message was new to them. The violence message was consistently the most believable, 89.4%, and personally relevant label message, 40.1%. The health myths were lowest for believability at 62.3%, followed by cancer message at 65.2%. Females believed violence 89.9% and the calories 81.4% more than males violence 89% and the calories 77.6% messages. Males believed in cancer messages more 65.8% compared to females 64.1%. Cancer message was found to be the highest for making them consider drinking less/changing behaviour as 39.6% reported; followed by the liver message, 31%. Females 44.2% compared to males 37% more likely responded to cancer, and also health myth, violence, and calories labels to consider their drinking reduction. For many of the messages, heavier drinkers reported that they would consider drinking less. Older respondents, compared to younger ones, believed nearly all the label messages. Over 25s stated that calories message would make them drink less, whereas under 25s preferred liver, heart, violence, cancer, and health myth messages. Overall, personal relevance was found to be really important for all messages; the text violence label (negative frame) was found to be the most believable and personally relevant, and the text cancer label (specific/positively framed) was most likely to make respondents consider drinking less. |
| Zhao et al. 2020 | Canada | Alcohol containers  Wine,  spirit,  beer,  cooler, liqueurs | Longitudinal study; An interrupted time series study quasi experimental design. | 15+ years | Aim: Evaluated whether the effects of the newly introduced alcohol warning labels would be associated with reduced alcohol consumption per capita for 28 months before and 14 months after the intervention; and two neighbouring regions control sites that already had pregnancy labels and driving/general label requirements.  Results: For labelled products, the consumption of alcohol was higher for males, younger respondents, and lower income levels. In the intervention site, there was a reduction in alcohol per capita sales from Whitehorse liquor stores, a 6.31% decrease compared to baseline (this was during the period of new warnings)- with per capita sales in Yukon's five regions, and also after adjustment for alcohol monthly sales in total per capita in neighbouring Northwest Territories. There was an increase in unlabelled sales during the study period. Per capita, sales decreased for labelled products by 6.59% and increased for unlabelled ones by 6.91%. During post labelling period, when pregnancy warnings were reintroduced, there was a reduction of 9.97%. There was only a reduction in alcohol consumption for those alcohol products with a red intervention and bright yellow labels, not those unlabelled. There was the greatest reduction in monthly sales after only the standard drink and low-risk drinking guidelines labels were reintroduced. Overall, there was an association with reduced per capita alcohol consumption. Media coverage of the study may have impacted the results as during the following three months, when there was a 5% reduction in sales; there was a lot of media coverage that could have reinforced the messages even though no new labels were added. Note continuation of Hobin et al. 2020a, Hobin et al. 2020b, Hobin et al. 2020c. |
